# Natural levee evolution in vegetated fluvial‐tidal environments

**DOI:** 10.1002/esp.5003

**Published:** 2020-10-01

**Authors:** Marcio Boechat Albernaz, Lonneke Roelofs, Harm Jan Pierik, Maarten G. Kleinhans

**Affiliations:** ^1^ Faculty of Geosciences, Department of Physical Geography Utrecht University Princetonlaan 8A Utrecht 3584 CB The Netherlands

**Keywords:** geomorphology, eco‐morphodynamic, levees, crevasses, deltas, estuary, vegetation

## Abstract

Natural levees are common features in river, delta and tidal landscapes. They are elevated near‐channel morphological features that determine the connection between channel and floodbasin, and consequently affect long‐term evolution up to delta‐scales. Despite their relevance in shaping fluvial‐tidal systems, research on levees is sparse and often limited to fluvial or non‐tidal case studies. There is also a general lack of understanding of the role of vegetation in shaping these geomorphic units, and how levee morphology and dimensions vary in the transition from fluvial to coastal environments, where tides are increasingly important. Our goal is to unravel the effects of fluvial‐tidal boundary conditions, sediment supply and vegetation on levee characteristics and floodbasin evolution. These conditions were systematically explored by 60 large‐scale idealized morphodynamic simulations in Delft3D which self‐developed levees over the course of one century. We compared our results to a global levee dataset compilation of natural levee dimensions. We found that levee height is determined by the maximum water level, provided sufficient levee building sediments are available. Discharge fluctuations increased levee width and triggered more levee breaches, i.e. crevasses, that effectively filled the fluvio‐tidal floodbasin. The presence of wood‐type (sparse) vegetation further increased the number of crevasses in comparison with the non‐vegetated scenarios. Conversely, reed‐type (dense) vegetation strongly dampened tidal amplitude and reduced the accommodation space and sedimentation further into the floodbasin, resulting in narrower levees, no crevasses and limited floodbasin accretion. However, dense vegetation reduced tidal forces which allowed levee growth further downstream. Ultimately, the levees merged with the coastal barrier, eliminating the floodbasin tides entirely. Our results elucidate the mechanisms by which levee and crevasse formation, and vegetation may fill fluvio‐tidal wetlands and affect estuary evolution. This brings new insights for geological reconstructions as well as for the future management of deltas and estuaries under sea‐level rise. © 2020 The Authors. Earth Surface Processes and Landforms published by John Wiley & Sons Ltd

## Graphical abstract

We self‐developed levees, crevasses and floodbasin evolution in fluvial‐tidal environments over a century using Delft3D morphodynamic numerical model. Our results unravel the main effects of fluvial‐tidal discharges, sediment supply and vegetation on the geomorphology. Our findings have implications for geological reconstructions and future forecast of estuaries and deltas in view of sea level rise and delta management.

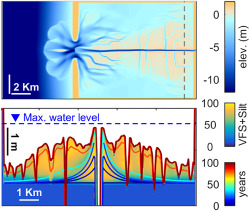

## Introduction

**Figure 1:**
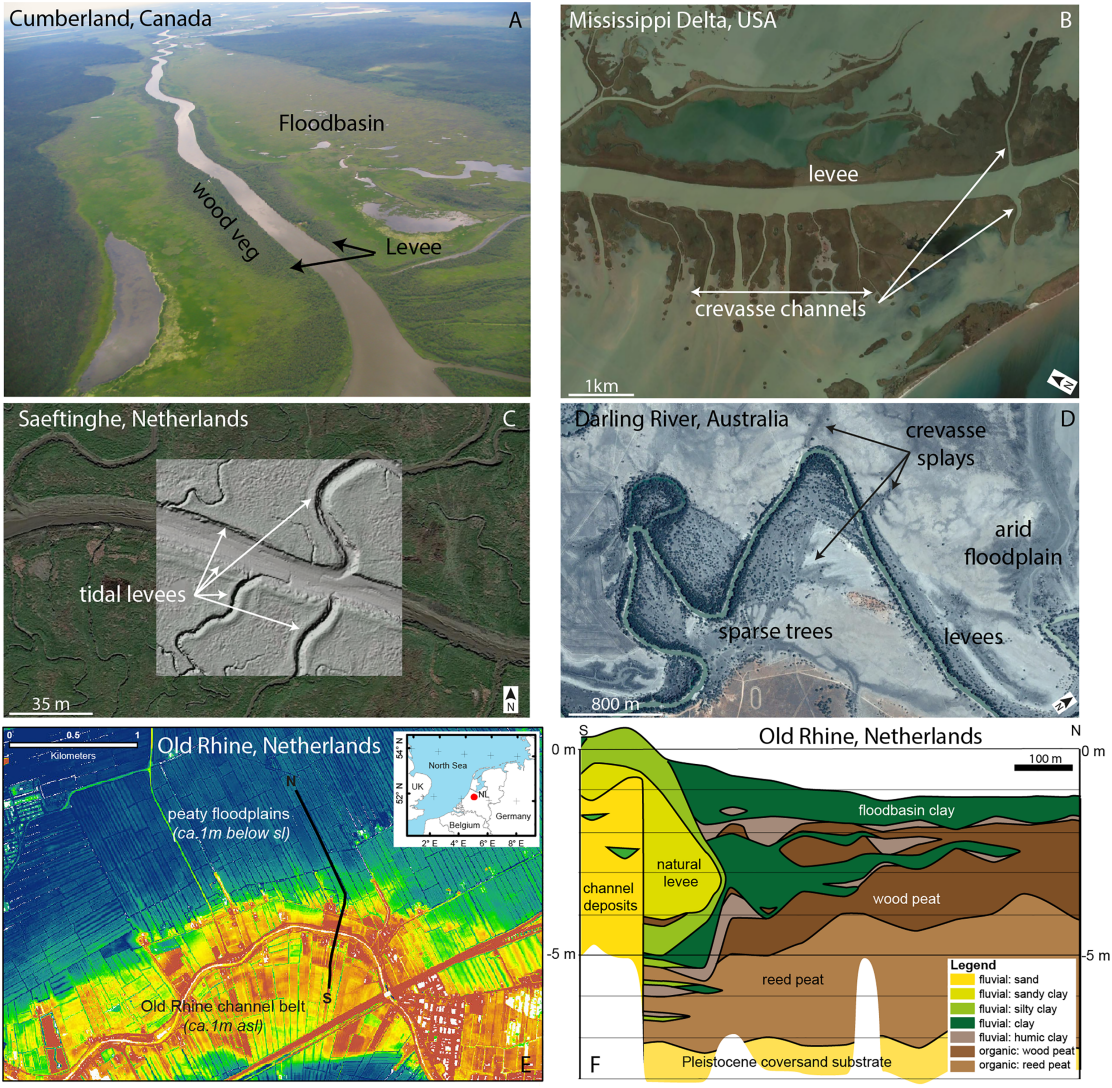
Overview of levees. (a) Levee complex at Cumberland, Canada ‐ aerial photos by MGK. (b) Levees and crevasses at the lower Mississippi Delta, USA ‐ from Google Earth imagery. (c) Tidal levees at Saeftinghe marsh, the Netherlands ‐ color image from Google Earth and hillshade from AHN www.ahn.nl. (d) Levees and crevasses splays of Darling River on the arid environment of Australia ‐ from Google Earth imagery. (e) Elevation map of the Old Rhine, NL from AHN www.ahn.nl. (f) Geological cross‐section of the Old Rhine after Stouthamer (2001). [Colour figure can be viewed at wileyonlinelibrary.com]

Natural levees, hereafter called levees, are along‐channel elevated areas (Figure 1) that slope towards the adjacent floodbasin (Brierley *et al*. 1997). Levees are found in both fluvial and tidal environments, commonly associated with vegetation, and are formed by differential sedimentation between the active channel and floodbasin (Cazanacli and Smith, 1998). Levees control the distribution of water, nutrients and sediment onto the alluvial valley and delta (e.g. Hiatt and Passalacqua, 2015). Over the course of their lifetime, levees can experience several breaches, called crevasses. Crevasses tend to be temporary features as they silt up, unless the breach turns into a major avulsion or bifurcation (Slingerland and Smith, 1998; Törnqvist and Bridge, 2002; Kleinhans *et al*. 2013; Nienhuis *et al*. 2018). The formation of crevasses leads to effective distribution of water and sediment further into the floodbasin even after the levee has built up to flood levels. Understanding how levee and crevasse morphodynamics affect the geomorphic evolution of fluvial‐tidal landscapes is vital for long‐term management of deltas and estuaries in view of sea level rise and human interventions, and elucidating previous and future sediment budgets.

The definition of a levee, although straightforward, is difficult to be systematically applied between geomorphologists and geologists (Brierley *et al*. 1997). In general, levees are along‐channel elevated areas which comprise intermediate sized sediments (e.g. very fine sand and silt) and are located between coarse active channel sediments and the fine floodbasin fill (Cazanacli and Smith, 1998; Makaske *et al*. 2002; Adams *et al*. 2004; Smith and Pérez‐Arlucea, 2004, 2008; Filgueira–Rivera *et al*. 2007; Burns *et al*. 2019). Geomorphologists tend to define a critical slope for levee definition (Cazanacli and Smith, 1998), whist for geologists overbank lithology is a more commonly followed criterion. This is because in ancient geological strata differential compaction subsequent to deposition may result in deviations from the original levee slope (Stouthamer, 2001; Burns *et al*. 2019; Pierik *et al*. 2017). However, both definitions have practical limitations to clearly identify the transition between levee and floodbasin from field records, especially for quantitative assessments.

Levee research has so far largely focused on case studies of fluvial environments through geological and morphological field data (e.g. Umitsu, 1985; Cazanacli and Smith, 1998; Makaske *et al*. 2002; Adams *et al*. 2004; Smith and Pérez‐Arlucea, 2004, 2008; Filgueira–Rivera *et al*. 2007; Johnston *et al*. 2019), modelling studies of only hydrodynamics in jet and mouth bar configurations (Mariotti *et al*. 2013; Canestrelli *et al*. 2014), or small scale (experimental) morphology (e.g. Rowland *et al*. 2010). These previous works either cannot isolate the effects of individual variables from the field, or in the case of modelling, do not account for the complexity of morphodynamic feedbacks in the scale of a fluvial‐tidal system. The field studies demonstrate a variety of possible levee shapes, slopes and total volumes, (Cazanacli and Smith, 1998; Adams *et al*. 2004; Gibling, 2006; Pierik *et al*. 2017) even across short distances along an individual channel. What causes this variability is presently uncertain due to scarce available data and difficulties in isolating the effects of internal and boundary conditions from field observations, especially the presence and effects of vegetation. While vegetation is abundant in fluvial and estuarine environments (e.g. Cazanacli and Smith, 1998; Corenblit *et al*. 2011; Pierik *et al*. 2017; Temmerman *et al*. 2007, and Figure 1), vegetation effects on hydrodynamics and morphology are often disregarded and understudied despite their strong influence on morphodynamics (D'Alpaos *et al*. 2007; Kirwan and Murray, 2007; Temmerman *et al*. 2007; Davies and Gibling, 2011; van Maanen *et al*. 2015; Kleinhans *et al*. 2018; Lokhorst *et al*. 2018; McMahon and Davies, 2018; Brückner *et al*. 2019). This paper aims to illustrate the growth processes, limiting factors and morphodynamic feedback of levee formation in fluvial‐tidal environments, including the presence and effects of vegetation.

Levee incipience and growth occurs when water level exceed channel heights and induce overbank discharge. This overflow is controlled by river discharge, sometimes in combination with tides. Coarser sediments are deposited in proximity to active channels with finer materials grading into more distal reaches of the floodbasin (Cazanacli and Smith, 1998; Adams *et al*. 2004). In the incipient growth stage, levees tend to be narrow and steep (Filgueira–Rivera *et al*. 2007). The progressive growth in height diminishes the transport of coarser material over channel banks. In this later stage, levees tend to widen with finer material (silt and clay) towards the floodbasin, which reduces the overall levee slope (Filgueira–Rivera *et al*. 2007) and generally creates an fining upward sequence (Törnqvist and Bridge, 2002; Pierik *et al*. 2017; Burns *et al*. 2019).

Our lack of understanding of the importance of boundary conditions in levee formation means, for example, that we do not know whether fluvial‐tidal levees develop relatively larger (or faster) than fluvial levees. We also do not know how levees and vegetation influence the fluxes of water and sediment distribution between channel and floodbasin, which determines long‐term delta development.

We hypothesize that the interplay between fluvial and tidal boundary conditions, sediment supply and vegetation are key in determining the end‐member morphology of levees‐crevasses and floodbasins. We aim to understand levee‐crevasse formation and floodbasin evolution, including the so far understudied effects of tides and floodbasin vegetation, in addition to variations in fluvial discharge and sediment supply.

We performed long‐term (*i.e*. 100 years) idealized numerical simulations of an entire coastal‐fluvial system using a morphodynamic model (Delft3D). In total, 60 scenarios were simulated under varying fluvial discharge, tidal amplitude, sediment concentration and under the effects of two vegetation types: reeds (dense) and trees (sparse). The model encompasses six sediment fractions grading from coarse sand to clay, and provides detailed stratigraphy and sediment sorting. With this approach we aim to self‐develop levees, covering key end‐member environments from nature. Our results are then compared to a large database of measured natural levees (compilation available in the supplementary material). Our model setup and scenarios were largely inspired by the Saskatchewan and Columbia Rivers in Canada (Cazanacli and Smith, 1998; Adams *et al*. 2004; Smith and Pérez‐Arlucea, 2008), and the ancient Old Rhine estuary in the Netherlands (de Haas *et al*. 2019). The channels and boundary conditions in the Canadian rivers have been studied extensively, and one important finding was that the supply of silt from the formerly glaciated hinterlands facilitated the rapid formation of high levees (Cazanacli and Smith, 1998; Perez‐Arlucea and Smith, 1999; Makaske *et al*. 2002; Adams *et al*. 2004; Filgueira–Rivera *et al*. 2007; Smith and Pérez‐Arlucea, 2008). The Old Rhine is a data‐rich fluvial‐tidal system which contains levees and crevasses that evolved throughout the Holocene from a tidal basin into a river estuary. This evolution was partially steered by upstream avulsions that rerouted the full Rhine river discharge into this branch between 6000 and 3000 years BP. After that, upstream avulsions progressively diverted discharge away from the Old Rhine, and wave‐induced sediment transport closed off the mouth (Berendsen and Stouthamer, 2000; Stouthamer, 2005; Cohen *et al*. 2012; Pierik *et al*. 2018; de Haas *et al*. 2019).

## Methods

The morphodynamic simulations were performed in Delft3D FLOW2D3D version 6.02.13.7658 from tag 7545 (Deltares, 2020). Delft3D is an extensively applied morphodynamic model of finite differences solving the momentum and continuity equations for unsteady shallow‐water flow in depth‐averaged mode through the Navier‐Stokes equation with hydrostatic pressure approximation (Deltares, 2017). The model computes accurate hydrodynamics and morphology, see Lesser *et al*. (2004), in addition to including the effects of vegetation on the hydrodynamics.

Below we detail the relevant model settings, including the initial and boundary conditions, and the basic data analysis.

### Model settings

**Figure 2:**
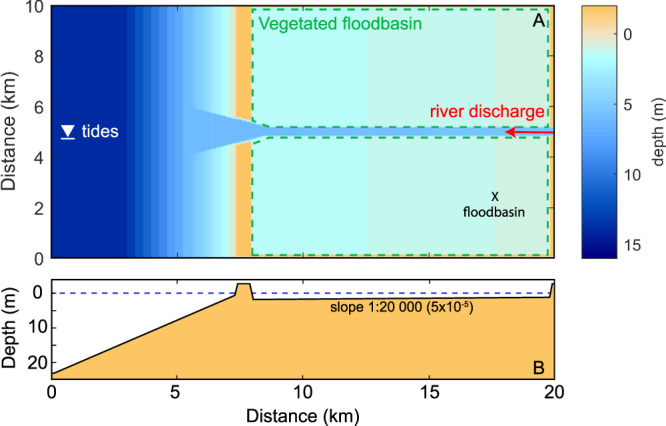
Delft3D model layout comprising initial bathymetry and boundaries. (a) plan view of model domain with boundary locations and the position of the floodbasin measurements denoted by the 'x' mark; (b) cross‐section view along the domain. [Colour figure can be viewed at wileyonlinelibrary.com]

The model domain consist of a 20 by 10km idealized estuarine environment (Figure 2) that provides enough time‐space for the levee‐crevasse development and the evolution of fluvial‐tidal landscape. The estuary comprises a 12.5 by 10km flood‐tidal basin enclosed by barriers, representing barrier islands, connected to a coastal zone of 7.5 by 10km on the seaside, while being fed by a river discharge on the landward upstream side of the basin.

The numerical simulations are depth‐averaged models (2DH) with 100 m resolution in the flow direction (along the river) and 50 m across. The domain contains a null gradient Neumann condition at the cross‐shore sea boundary, water level on the seaward side and river discharge on the upstream basin limit. An initial channel was carved across the basin connecting the upstream river to the inlet, ending with a divergent shape at the coast. The width and depth dimensions of the initial river channel were determined based on geometric relation ( $Q=whu\;[m^{3}/s]$ where *Q* is the river discharge; *w* the channel width; *h* the channel depth and *u* flow velocity) aiming for an initial flow velocity of 0.5 *m*/*s* and constant width‐depth ratio of approximately 45. For example, a scenario with 700 *m*^3^/*s* discharge consists of a channel with 250 m wide and 5.5 m depth dimensions. The basin slopes with 5*x*10^−5^ (*i.e*. 1:20,000), corresponding to approximately 0.6 m height difference between the inlet and river mouth. Six sediment fractions were deployed for simulating complex sediment sorting and allowing levee evolution out of a subset of these fractions for a wide range of flow conditions. The initial substrate contains four sand sizes ranging from coarse (300 μm) to very fine sand (75 μm). The two finest fractions, namely silt and clay material were supplied by a given concentration during the simulation via the upstream river boundary, while the other sand‐sizes are supplied by an equilibrium concentration boundary condition.

The model scenarios were systematically varied with different combinations of (1) time‐constant fluvial discharge magnitudes, (2) tidal amplitude, (3) fine sediment supply, (4) the time‐variable fluvial discharge, (5) sediment starvation of very fine sand and silt, (6) and the presence of two types of vegetation (Table 1 and Table A1). The time‐constant fluvial discharge magnitude varies among different runs between 400 *m*^3^/*s* and 1500 *m*^3^/*s*. The tidal amplitude of the M2 tidal component ranges between 0.5 and 1.25 m in steps of 0.25 m. Additionally, the M4 component was coupled with 10% of the M2 amplitude and 75 degrees phase lag, resembling the West (Holland) coast of the Netherlands on the North Sea. The fine sediment supply was delivered from the fluvial upstream boundary varying between 0 *g*/*m*^3^ and 20 *g*/*m*^3^ of equal amounts of clay and silt, resulting in a total mud concentration up to 40 *g*/*m*^3^. We also performed simulations without very fine sand and silt to assess if these were limiting factors for levee development in a sediment‐starved system. The influence of discharge variability was included with a yearly based peak discharge of different magnitudes. For comparison, the yearly integrated discharge was kept constant, at 700 *m*^3^/*s*, for all variable discharge scenarios.

**TABLE 1:** Overview of model scenarios and boundary conditions simulated in Delft3D. In total 60 scenarios (see Table A1) combined: (1) Discharge magnitude, (2) tidal amplitude, (3) total concentration of fines, (4) discharge variability, (5) absence of very fine sand and silt, (6) and the inclusion of two types of vegetation. The reference scenario (model 40) is highlighted in **bold**.

**Model Scenarios**	**Unit**	**Absent**	**Low**	**Medium**	**High**	**Very high**
1. Discharge magnitude	*m*^3^/*s*	‐	400	**700**	1000	1500
2. Tidal amplitude (M2)	m	0	0.25‐0.5	**0.75**	1.0	1.25
3. Mud Concentration	*g*/*m*^3^	0	10	**20**	30	40
	**Peak Q**	**Low Q**	**Mean Q**		
	*m*^3^/*s*	*m*^3^/*s*	*m*^3^/*s*		
4. Discharge variability	1000	692	700		
	1250	685	700		
	1500	678	700		
5. Sediment‐starved	no silt, no very fine sand					
6. Vegetation	Dense (reeds)			Sparse (trees)		

Finally, vegetation was included in the floodbasin (see Figure 2) as dense and sparse type of plants. The vegetation typologies resemble the parameters (Table 2) of reeds (dense) and trees (sparse), based on van Oorschot *et al*. (2017). The vegetation was simulated with the Baptist *et al*. (2007) formula which affects the morphodynamics in two ways: first, it computes a new bed roughness (C) accounting for the vegetation ensemble in each grid cell, as follows:

(1) $$C=\overset{\text{submerged}}{\overbrace{\underset{\text{emerged}}{\underbrace{C_{b}}}+\;\frac{\sqrt{g}}{\kappa }\;\ln\left(\frac{h}{h_{v}}\right)\sqrt{1+\frac{C_{D}\;n\;h_{v}\;C_{b}^{2}}{2g}}}}$$

where: *C* = Chézy value added with vegetation [*m*^0.5^/*s*]; *C*_*b*_ = base Chézy value [*m*^0.5^/*s*]; *C*_*D*_ = drag coefficient induced by vegetation [‐]; *n* = vegetation density [1/*m*]; *h*_*v*_ = vegetation height [*m*]; *h* = water depth [*m*]; *g* = gravity acceleration [*m*/*s*^2^]; *κ* = von Kármán constant [‐]; second, it introduces a drag force into the hydrodynamics as $\frac{\lambda }{2}u^{2}$, coupled into the momentum equation:

(2) $$\lambda =\overset{\text{submerged}}{\overbrace{\underset{\text{emerged}}{\underbrace{C_{D}\;n}}\;\frac{h_{v}\;C_{b}^{2}}{h\;C^{2}}}}$$

[Correction added on 20 November 2020, after first online publication: Presentation of equations 1 and 2 is previously incorrect and has been updated in this version.]

where, *λ* = flow resistance due to vegetation [1/*m*]; *u* = flow velocity [*m*/*s*]. Therefore, the vegetation also affects the flow, via an additional drag force term *λ*, instead of solely increasing the bottom roughness which would lead to overprediction of sediment transport rates due to the increase of the bed shear stress. More details regarding the implementation of vegetation can be found in Baptist *et al*. (2007) and Deltares (2017).

**TABLE 2:** Vegetation parameters used as model inputs to simulate dense and sparse vegetation. *n* is vegetation density, *h*_*v*_ is vegetation height, *C*_*b*_ = Chézy value with considering vegetation, *C*_*D*_ = drag coefficient and *Area* is the coverage percentage.

	n	*h*_*v*_	*C*_*b*_	*C*_*D*_	Area
units	1/*m*	m	*m*^0.5^/*s*	‐	%
**sparse**	0.05	3	45	1.2	0.5
**dense**	3	3	45	1.0	0.3

In order to have a frame of comparison between the models, we elected the typical scenario (model 40) as our reference model run, with 700 *m*^3^/*s* fluvial discharge, 0.75 *m* tides and 20 *g*/*m*^3^ of mud (clay & silt).

We selected the sediment transport predictor TRANSPOR2004 (van Rijn *et al*. 2004; van Rijn, 2007a, 2007b) because it is well‐calibrated on a wide range of environments, including tidal‐fluvial conditions. It conceptually separates bed and suspended load and allows calculations with multiple sediment size fractions. In Delft3D the mud fractions are treated as cohesive sediments and the deposition and erosional fluxes are computed according to Partheniades‐Krone formulation (Partheniades, 1965) based on user‐defined critical shear stresses. The erosion shear stress was set to 0.5 *N*/*m*^2^ and the sedimentation threshold to 1000 *N*/*m*^2^ (the high value means that it always allow for sedimentation), both default values. The transverse bed slope sediment transport was parameterized with Koch and Flokstra (1981) to have less morphological diffusion than Ikeda (1982), after Baar *et al*. (2019), wherein the sediment transport vector is rotated downslope as a function of transverse slope divided by *α* ∗ *θ*^*β*^, where *θ* is the sediment mobility, and here α=0.2 and β=0.5.

The stratigraphic bed module from Van Kessel *et al*. (2012) was used to allow different sediment mixtures and the effect of differential bed composition on sediment transport rates for each sediment fraction. The module tracks and saves the bed composition with a user‐defined vertical resolution, here 10cm, and the sediment transport is computed for the active top‐layer on the basis of the top sediment mixture. With this approach, we were able to represent the sediment dynamics of different sub‐environments, for example channel, floodplain and levees, similar to van der Vegt *et al*. (2016).

A constant morphological acceleration factor (morfac) of 200 was used to speed up the simulations and 100 morphological years were performed in total. Preliminary runs (not shown) demonstrated the limited effects of the acceleration factor on the final morphology, in agreement with Ranasinghe *et al*. (2011), whilst the high computational cost of multiple size fractions and one century development required a high morfac. The variable discharge scenarios included a time‐varying morfac to incorporate the higher discharges by means of lower acceleration factors, here 20, during peak discharges.

Comprehensive model settings are specified in the supplementary material.

### Data analysis

**Figure 3:**
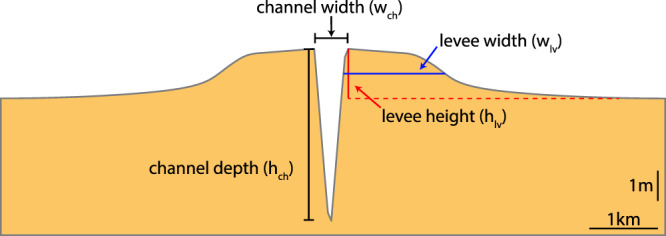
Discrimination of main levee and channel dimensions applied during analysis. This representative cross‐section corresponds to the final levee of model 38. Levee height and width are quantified as the average between both sides of the levee. [Colour figure can be viewed at wileyonlinelibrary.com]

We quantified levee dimensions based on their morphology, similar to e.g. Cazanacli and Smith (1998); Adams *et al*. (2004); Filgueira–Rivera *et al*. (2007). Levee height and width were extracted from the most upstream 5km of the floodbasin portion in order to avoid the disturbance by the main tidal channel network. The representative levee profile is the average elevation on the longitudinal direction along this section. Levee height was computed as the largest prominence in the cross‐section, while the width corresponds to the lateral extent of half a levee height (Figure 3). The final value for height and width is the average between both sides of the floodbasin. The channel depth and width correspond to the bankfull channel depth (i.e. from the bottom of the channel up to the levee height) and bankfull channel width (i.e. distance between the two levee crests), respectively.

Crevasse channels were counted along the central channel whenever the breach reached more than 0.5 m depth in the main levee. For counting the number of crevasses, we considered the entire reach between the barrier and the upstream river, instead of restricting to the 5 km upstream reach, up to the point where the main levee could be identified.

## Results

### Levee development under fluvial‐tidal conditions

**Figure 4:**
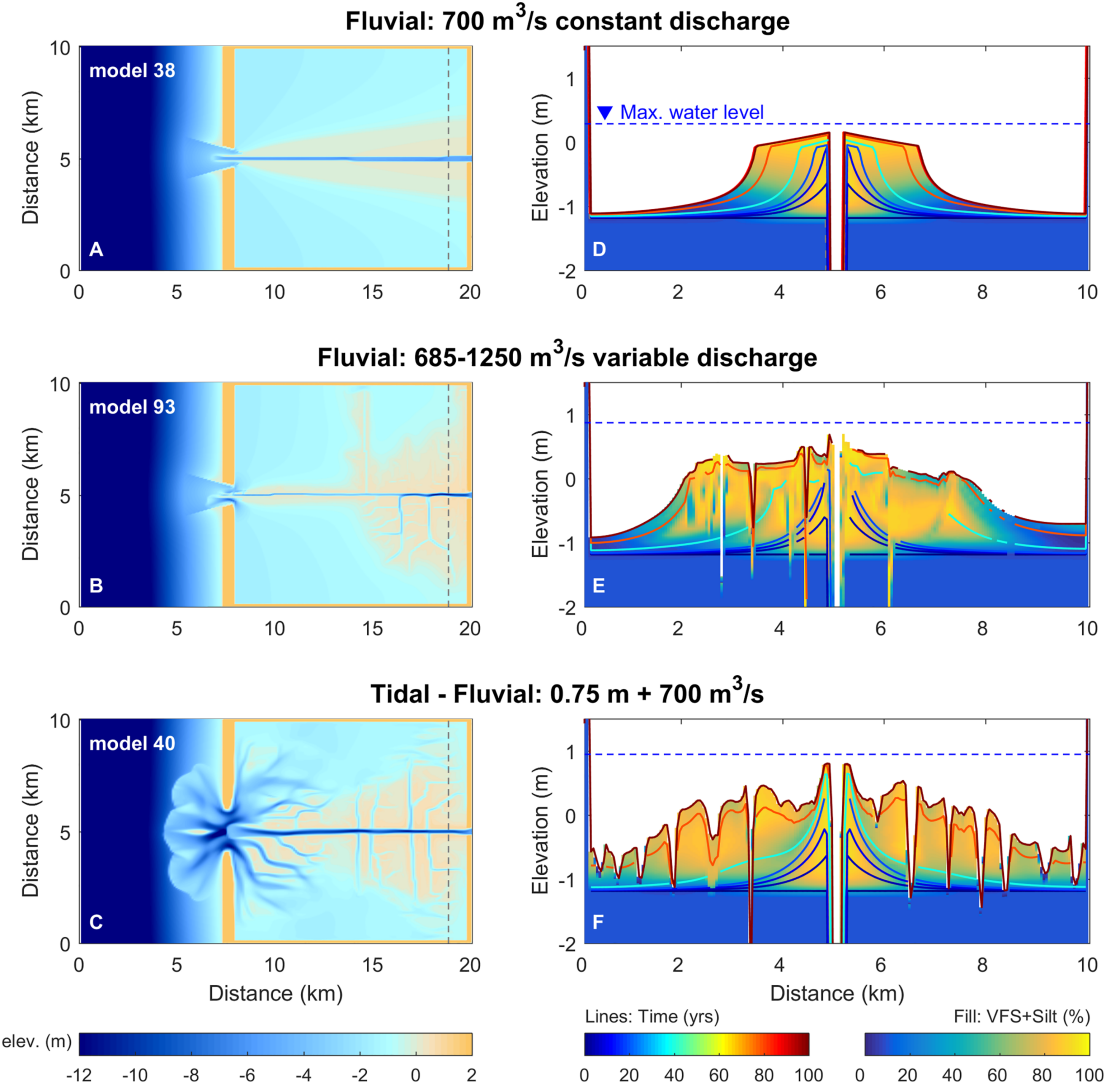
Levees after 100 years under a range of fluvial‐tidal boundary conditions. Left panels (a‐c) show the final plain‐view morphology. Right panels (d‐f) show the cross‐section, illustrated in the correspondent map on the left, with combined percentages of very fine sand (VFS) and silt in the colored fill scale and the bed level evolution shown in colored lines. [Colour figure can be viewed at wileyonlinelibrary.com]

Levee development predominantly starts with vertical growth towards the maximum water level, followed by the lateral expansion into the floodbasin (Figure 4). The dimensions are larger near the upstream boundary, which is the main source of sediments, and decrease in size and volume towards the downstream portion of the domain. Levees are mainly formed by silt and very fine sand (VFS) along the main channel. The coarser sand fractions are dominant within the active main channel, while clay generally settles further into the floodbasin. The deposits near the channel are relatively coarser, fining upwards and laterally.

Distinct geomorphological patterns formed among the scenarios of time‐constant fluvial discharge (model 38), time‐varying fluvial discharge (model 93), and combined fluvial‐tidal discharge (model 40). In the absence of discharge fluctuations, levees are smooth and continuous (Figure 4,a) with a clearer transition from levee to floodbasin deposits (i.e. distal clays in Figure 4,d). In contrast, water level fluctuations, especially those induced by tides, trigger more crevasses (Figure 4,b,c) and enhance sediment mixture between the levee and floodbasin deposits (Figure 4,e,f), making the units and transitions among levee, floodbasin and tidal channels nearly indistinguishable in the lithological record.

**Figure 5:**
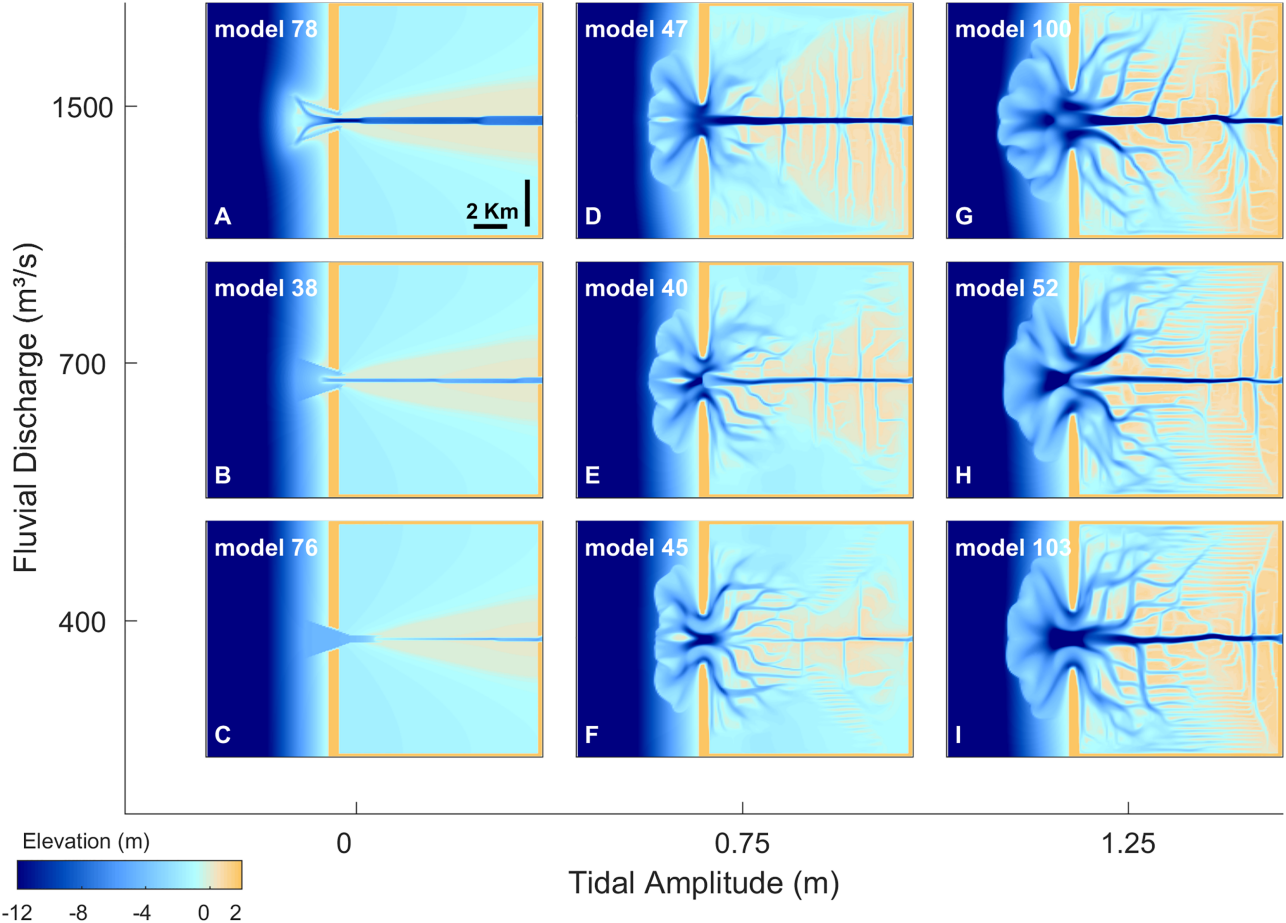
Morphological evolution after 100 years for combinations of fluvial discharge and tidal amplitude. [Colour figure can be viewed at wileyonlinelibrary.com]

As levee morphology and floodbasin evolution is considerably different after varying the fluvial‐tidal boundary conditions (Figure 4), we simulated a wide range of scenarios (Figure 5) varying the relative force between fluvial and tidal discharges (Table 1). Both the channel and its associated levees became wider when subjected to increased mean fluvial discharge. No crevasses were formed under the constant discharge scenarios (Figure 5,a‐c). Conversely, fluvial‐tidal conditions show abundant crevasse systems, together with tidal channels in the downstream portion of the basin closer to the inlet, and along the side flanks towards the upstream basin (Figure 5,d‐i). When both fluvial and tidal discharge are increased, the basin fills with more sediment and develops larger and more complex crevasses and more extensive tidal channel network (see Figure 5,g). The tidal channels tend to follow E‐W direction along the basin, while crevasses are oriented N‐S across the basin. The relative importance of fluvial versus tidal discharge leads to either more tidal or fluvial dominated morphology, which compete for space within the basin. Apart from the spatial dominance between tides and river, the general development of levees, channels and crevasses is rather similar across all scenarios (Figure 5,d‐g). In contrast, the models with time‐constant fluvial discharge developed no crevasses, but instead developed continuous and unincised levees (Figure 5,a‐c). We can therefore conclude that discharge fluctuations create an increasingly diverse morphology and deposits with larger crevasses and more complex channel networks.

### Effects of vegetation and sediment starvation

**Figure 6:**
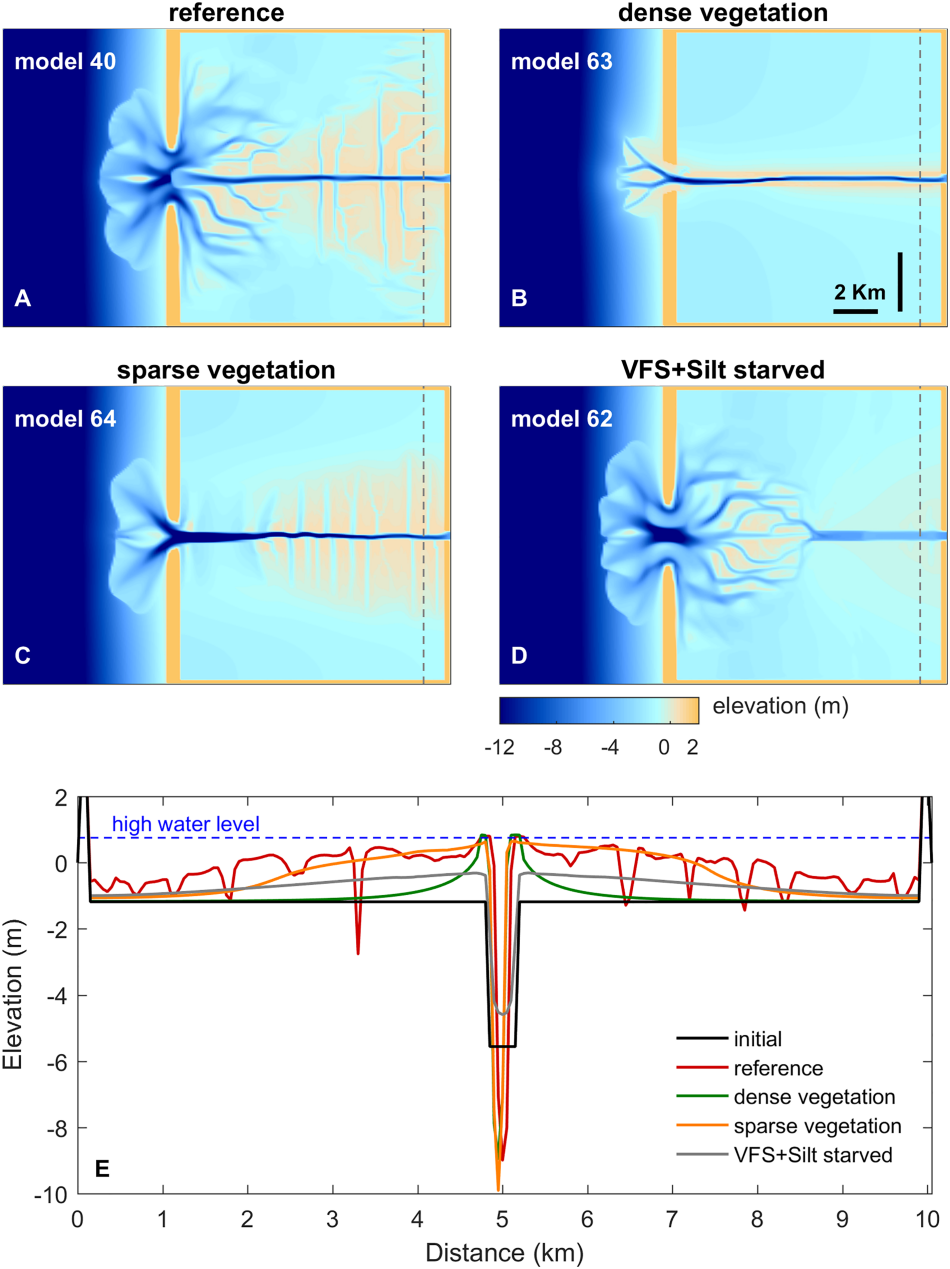
Effects of sediment starvation and vegetation on levee and floodbasin evolution. (a‐d) Plain view of described models. (e) Cross‐section of each model along the gray line in the top models. [Colour figure can be viewed at wileyonlinelibrary.com]

We introduced variations to the previous models regarding levee building sediment supply and inclusion of vegetation on the floodbasin. Starting from the reference fluvial‐tidal model (model 40, Figure 6,a) we included sparse (model 64, Figure 6,c) and dense (model 63, Figure 6,b) vegetation in the floodbasin, in addition to removing very fine sand and silt from the system (model 62, Figure 6,d).

The sediment‐starved scenario shows that without intermediate sediment sizes there is no levee formation and the near‐channel deposits do not grow in height towards the maximum water level, as they do in other scenarios. Without levees, clay gradually fills the floodbasin in a homogeneous gently sloping deposit initiating from the main channel (Figure 6,e).

**Figure 7:**
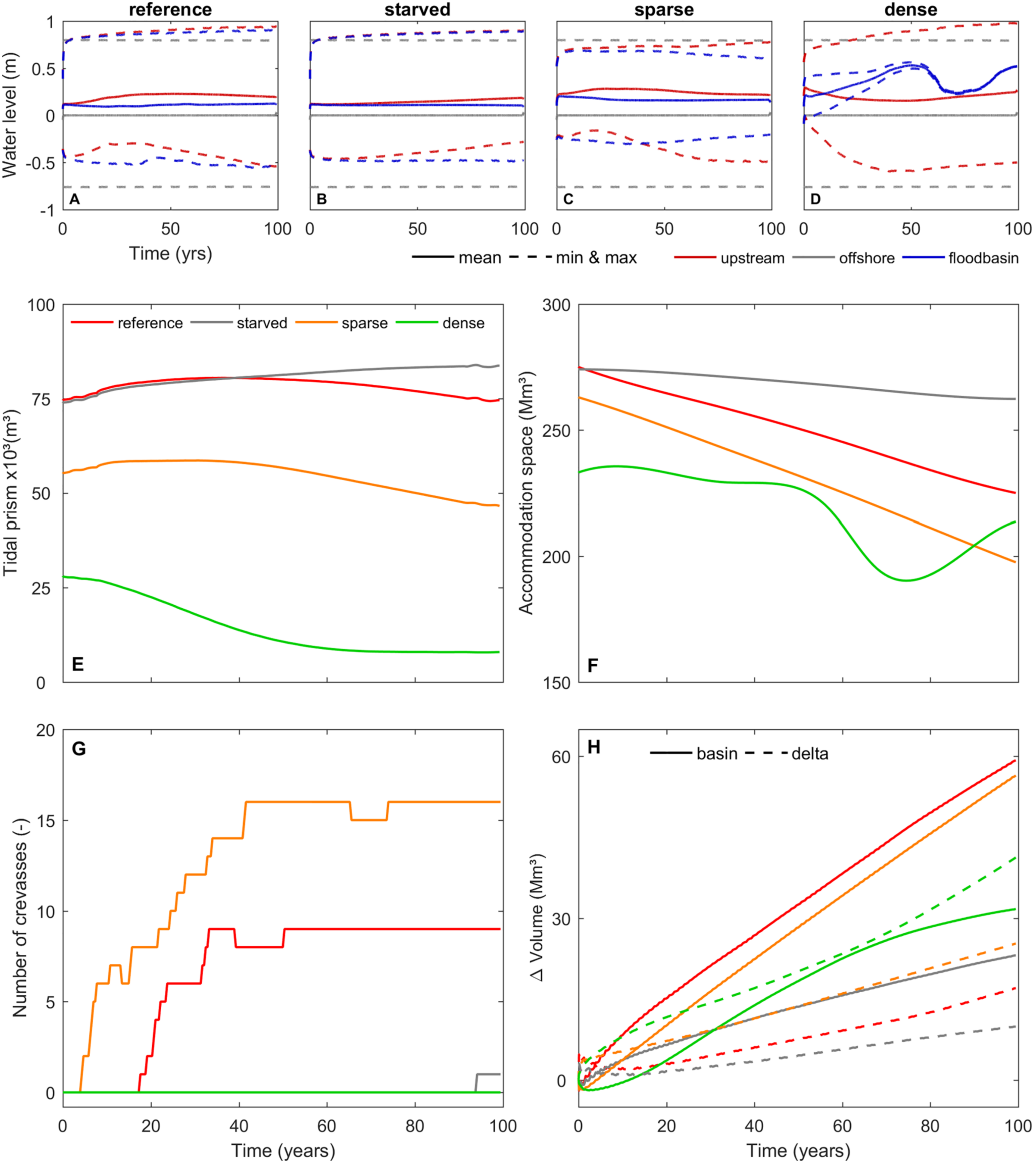
(a‐d) Comparison of water levels in the upstream fluvial river, floodbasin and the offshore tidal range. Models shown are the same as in Figure 6. The position of the floodbasin measurement location is shown in Figure 2. (e) Computed tidal prism and (f) accommodation space through time. (g) Number of crevasses along both sides of the main levee. (h) The sediment volume gain in the floodbasin and in the ebb delta. [Colour figure can be viewed at wileyonlinelibrary.com]

The effect of vegetation is also pronounced on the levee and floodbasin development. The dense vegetation (reeds) creates narrower and steeper levees compared to other scenarios (Figure 6,e). The main effects of dense plants are: (1) reducing the propagation of tidal flow into the basin and the resulting tidal prism (Figure 7,d,e), (2) inhibiting both crevasse formation (Figure 7,g) and lateral levee growth, and (3) leaving the basin sediment‐starved (Figure 7,h) as fluvial sediments are mainly exported to the ebb delta rather than stored in the basin. In the dense vegetation scenario, the narrow levee progressed from the upstream river as far as the coastal barrier, connecting the main channel to the open coast after approximately 75 years (Figure 6,b). This merging of levees and the coastal barrier ultimately inhibits the tidal propagation and isolates the floodbasin from fluvial‐tidal dynamics, and thus it resembles a fluvial dominant system despite the presence of offshore tides. With the levees fully connected to the coastal barrier, the floodbasin turned into a near‐stagnant water reservoir with higher mean water level in comparison to the main channel (Figure 7,d) and currents are almost entirely absent inside the basin. This water level gradient together with the reduced sediment dynamics inhibited the formation of crevasses and the basin infilling. Sparse (trees) vegetation (1) reduces the tidal propagation into the basin to a lesser extent, (2) facilitates more crevasses along the levee, (3) inhibits the formation of tidal channels, although tides still penetrate the floodbasin generating water level fluctuations and currents (Figures 6,c and 7).

In short, our scenarios demonstrate that no levee forms without the presence of specific sediment fractions, despite suitable boundary conditions and accommodation space. Vegetation creates higher but narrower levees. The models with sparse or no vegetation (reference scenario) induced more crevasses and trapped more sediment into the basin compared to the dense and sediment starved cases. The dense vegetation scenario was the only case where the sediment volume exported to the ebb delta topped the volume trapped inside the floodbasin (Figure 7,h).

**Figure 8:**
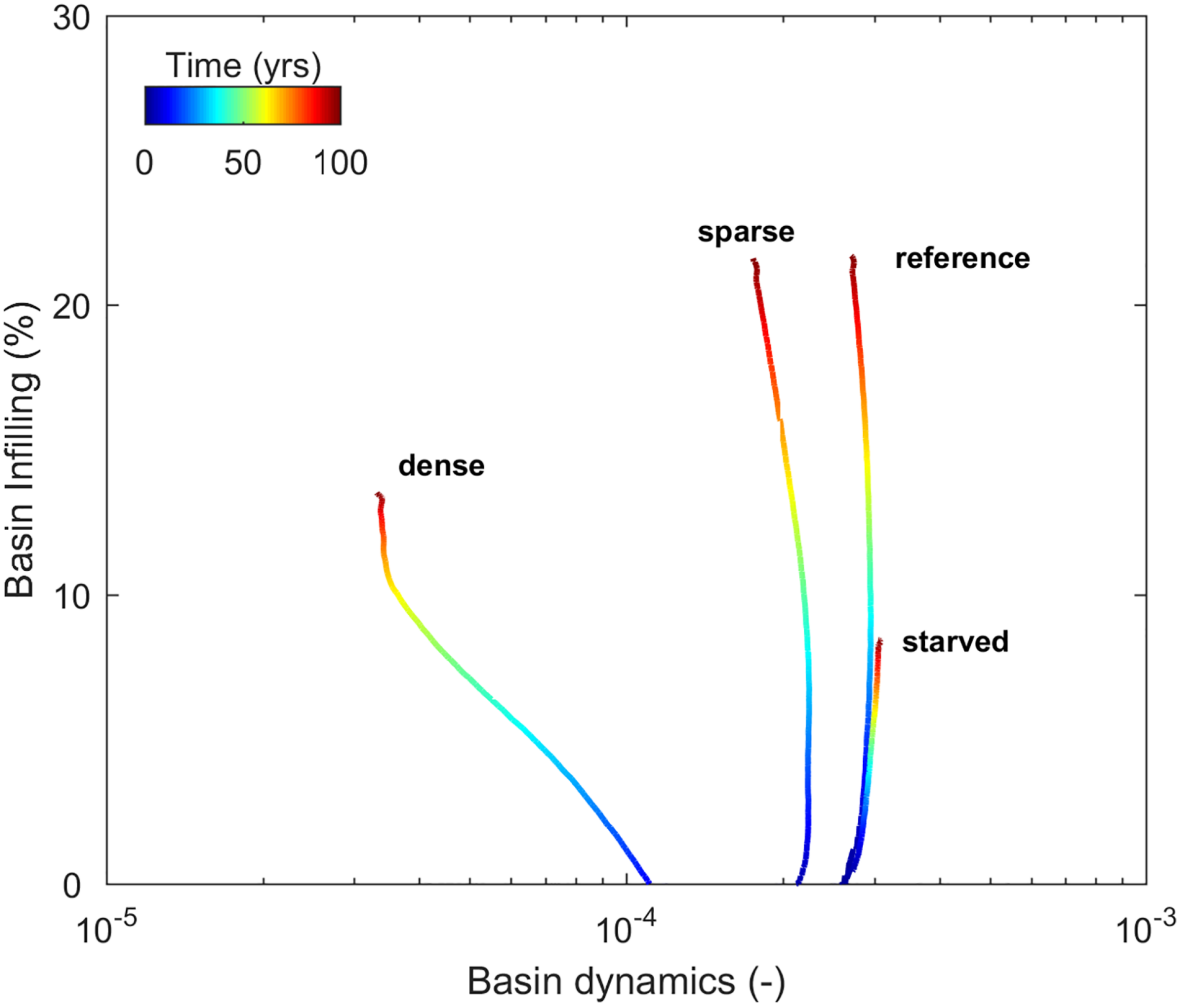
Basin dynamics versus basin infilling over time. Here the basin dynamics is defined as the tidal prism divided by the accommodation space, and basin infilling is the delta volume of deposited sediment divided by the initial accommodation space. Models shown are the same as in Figure 6. [Colour figure can be viewed at wileyonlinelibrary.com]

Another remarkable effect of vegetation is the reduction of tidal prism and accommodation space. We compute accommodation space as the total water volume within the floodbasin, and tidal prism as the water discharge through the inlet during one tidal cycle. For the same fluvial‐tidal boundary conditions, we observe a reduction of the initial tidal prism and accommodation space with the inclusion of vegetation on the floodbasin (Figure 7e,f). Through time, both accommodation space and tidal prism decrease as the basin progressively fills with sediment (Figure 7h). Hence, we expect different equilibrium states between the basin (hydro)dynamics and the import and export of sediment, for each scenario, after the initial morphological development. To test this hypothesis, we computed the basin dynamics, defined as the tidal prism divided by the accommodation space, as a proxy of how large and dynamic the basin is. Larger basin dynamics values indicate higher flushing per basin volume, while lower dynamics indicate a more stagnant condition. We plotted basin dynamics against the infilling percentage (defined as the sediment volume gain divided by the initial accommodation space) (Figure 8). The sediment‐starved and reference scenario, without vegetation, are the most dynamic cases with similar values. The starved scenario is slightly more dynamic than the reference case and does not show a decrease through time. Despite their similarity, those two cases have distinct infilling rates, as expected by the difference in sediment input. In contrast to the non‐vegetated scenarios, the sparse vegetated model shows less dynamics with the same infilling percentage with respect to the reference case. Their behavior also display a similar evolution with increasing basin dynamics at the beginning, before decreasing as the model runs. Conversely, the dense vegetation shows a constantly decreasing dynamic from initiation. After approximately 75 years, and after the levee connected with the barrier, the system finds a new equilibrium accompanied with lower infilling rate as sediments are predominantly carried out to the ebb delta at this later stage (Figure 7h). We conclude that vegetation reduces the tidal propagation and thus the basin dynamics, with dense vegetation having the strongest effect, ultimately inhibiting all tidal penetration inside the basin.

### Levee dimensions and evolution

**Figure 9:**
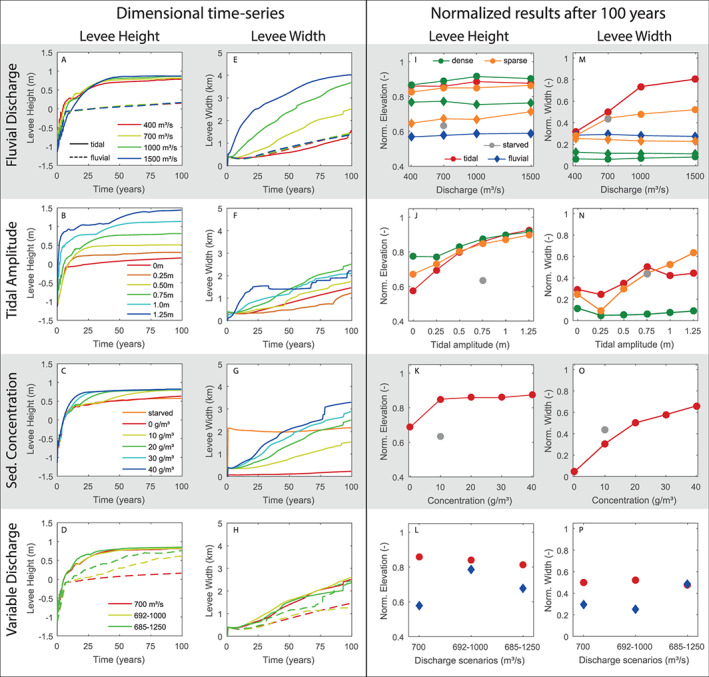
Dimensional time‐series of levee height and width (left two columns) and normalized results after 100 years (right two columns) for all 60 runs. Levee height was normalized by the maximum recorded water level and levee width was normalized by floodbasin width. The first row (a,e,i,m) shows variations in fluvial discharge with 0.75 m tides (solid lines; circles) and without tides (dashed lines; lozenge). The second row (b,f,j,n) shows varying tidal amplitude combined with 700 *m*^3^/*s* fluvial discharge. The third row (c,g,k,o) shows varying mud concentration, combined silt and clay, for 0.75 m tides and 700 *m*^3^/*s* fluvial discharge. The fourth row (d,h,l,p) shows variable discharge cases with 0.75 m tides (solid lines;circles) and without tides (dashed lines; lozenge). On the normalized cases (right side) vegetation is depicted in orange (sparse) and green (dense) while the starved cases, without VFS and silt, is shown in gray. [Colour figure can be viewed at wileyonlinelibrary.com]

We retrieved levee height and width from all 60 model scenarios (see Tables 1, A1) according to Figure 3. In addition, we normalized the final levee height and width after 100 years (Figure 9). Levee height was normalized by dividing the levee elevation by the maximum water level, and levee width was divided by the floodbasin width. This normalization indicates how much the levee grew towards its maximum possible dimensions (i.e. maximum water level and floodbasin width). As levees grow towards the maximum water level, tides and floods are strong controls on levee height, and sediment concentration to a lesser extent (Figure 9a‐d). After the initial predominantly heightening phase, levees start to widen. Levee width shows a more constant growth through time, strongly related to fluvial discharge and sediment concentration. In general, no levees are formed in absence of very fine sand and silt (Figure 6d,e). In this case, mud spreads all over the floodbasin, without a distinct near‐channel elevated ridge but instead a low, gently sloping very wide deposit that does not follow the maximum water levels. Hence, it is arguable that this deposit should not be classified as a levee. Despite this, we quantified the dimensions to compare it with all other scenarios.

The inclusion of vegetation creates higher but narrower levees, especially for the dense vegetated floodbasin. The sparse vegetation scenarios generate levees dimensions that fall between the dense and reference scenarios. Increasing the fluvial discharge, especially in combination with tides, causes levees to grow higher, (Figure 9a,e,i,m) whilst levee width has its maximum in mid‐tidal ranges as larger tides create more tidal channels in the basin that flank the levees (Figure 9b,f,j,n). Sediment supply only impacts levee height when there are very low concentrations, namely no mud (0 *g*/*m*^3^). However, levee width shows a strong relation with increasing mud concentration. Finally, we observe that fluvial discharge variations affect both height and width. Floods promote similar morphological effects to tides. Although, tides are more efficient in widening and heightening levees as the water level fluctuates once or twice a day while floods occur in a yearly time‐scale.

### Comparison with natural levees

Following the self‐development of levees from our numerical models, we compared our model results with measured data from several sites (Cazanacli and Smith, 1998; Latrubesse and Franzinelli, 2002; Makaske *et al*. 2002, 2007; Adams *et al*. 2004; Filgueira–Rivera *et al*. 2007; Funabiki *et al*. 2012; Klasz *et al*. 2014; Kiss *et al*. 2018) and extracted relations between the relevant channel and levee dimensions. Beyond a simple comparison for model validation, we intend to highlight and explore the main similarities and discrepancies between the model results and the measurements. Both measured and model data are available in the supplementary material.

**Figure 10:**
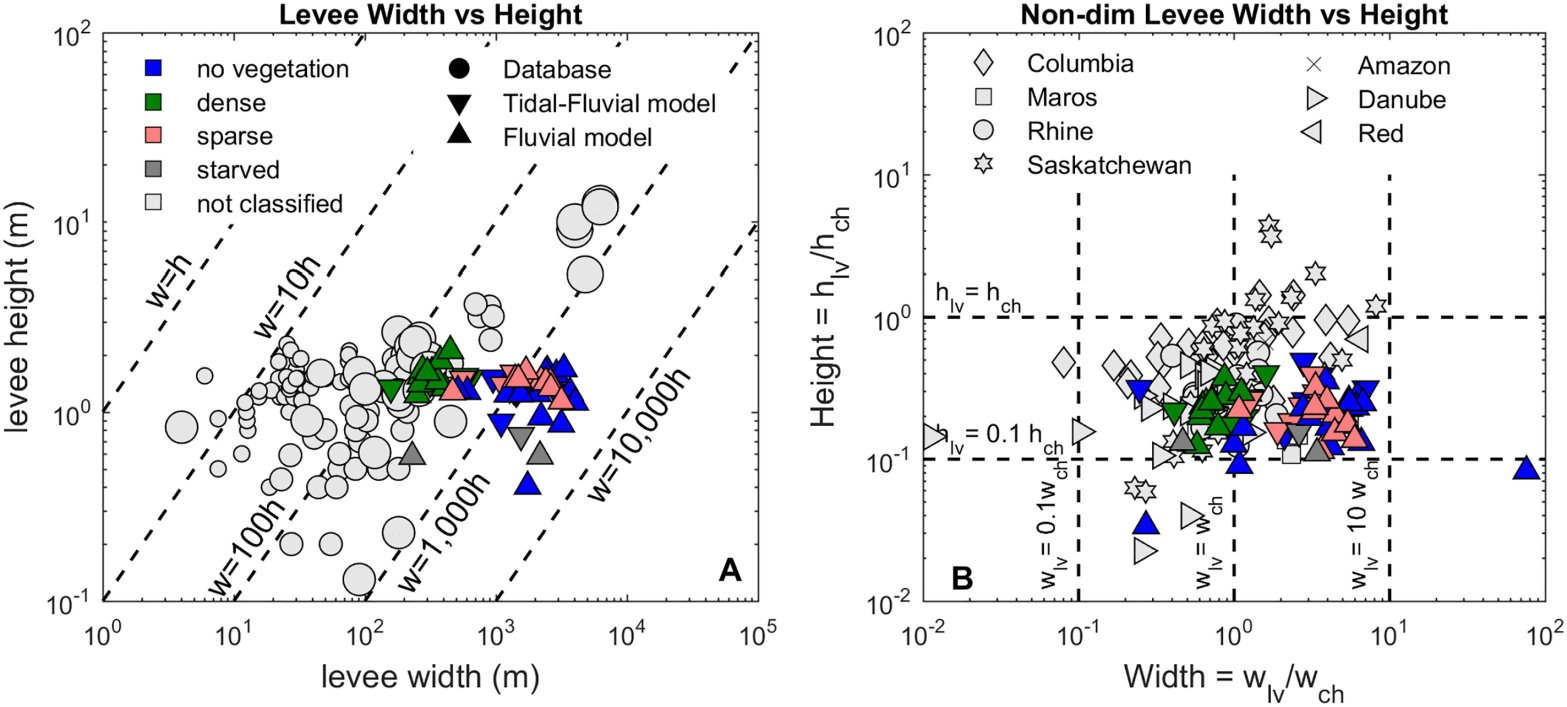
Comparison of levee height and width between model and dataset. The model scenarios are subdivided in tidal‐fluvial (downward triangle) and fluvial (upward triangle) and color coded by the scenario. The database is displayed in gray, i.e. not classified. (a) The dimensional levee height versus width where the sized symbols for the database (circle) represents mean fluvial discharge values. (b) The non‐dimensional height is the levee height divided by the bankfull channel depth and width is the levee width divided by the bankfull channel width. [Colour figure can be viewed at wileyonlinelibrary.com]

Modelled levee height (*h*_*lv*_) collapsed within the measured dataset range (Figure 10). Modelled levee width (*w*_*lv*_) from the dense‐vegetated scenarios also compared well with the measured levee dimension, while the sparse and non‐vegetated modelled scenarios overestimated levee width by approximately one order of magnitude. This mismatch in width between our models and the measured levees demonstrates the importance of vegetation in shaping morphology, and we address this topic more extensively in the discussion. Most natural vegetated levees have aspect ratios, calculated as width relative to height, between 10 to 100 times. The largest rivers, e.g. Amazon, Yellow River and Mekong, as well as our non‐vegetated cases can reach aspect ratios of up to 1000. Levee height varies from a few centimeters up to 3 m, again with the exception of the large rivers which reach up to tens of meters. Levees in our models varied between 0.4 and 2.1 m in height and 160 to 4025 m wide. We also see a similar trend when normalizing the levee dimensions by the channel dimensions. Most natural and model data have levee heights that correspond to ca. 20% of their channel depths. Levee width usually has the same order of magnitude of channel width, especially with dense vegetation. For the scenarios without vegetation and the fluvial‐tidal scenarios, we observe much wider levees, of around 4 times their channel widths.

The non‐dimensional levee data from the Columbia and Saskatchewan rivers show relatively high levees and shallow channels compared to our compilation of field and model dataset. These observations agree with the fact that these rivers are rich in silt, anastomosing and multi‐thread in pattern (Makaske *et al.* 2002), unlike the modelled scenarios and the other rivers from our database. The anastomosing character implies complex division of water and sediment discharges through the branches, which is relevant because individual channels tend fill over time following inactivation (Kleinhans *et al*. 2012). Hence, levee dimensions from the anastomosing Canadian rivers show larger scatter in respect of channel dimensions, while the Rhine, Maros, Amazon and Danube rivers data agrees relatively well with our model results.

In summary, the best agreement between the measured data and model results comes from the model scenarios including vegetation. The non‐vegetated scenarios result in wider levees that are rarely observed in nature nowadays. Those dimensions are comparable to the largest rivers in the world, however this apparent similarity derives from a much higher water and sediment discharge and not the lack of vegetation.

## Discussion

We self‐developed levees and crevasses in a morphodynamic model under comprehensive fluvial‐tidal boundary conditions, including the effects of vegetation and broad sediment composition and sorting. With our novel set of scenarios, we unraveled the most important conditions that control levee height and width and the formation of crevasses, and showed how vegetation alters levees, basin dynamics and consequently the overall landscape evolution. Now, we discuss the main findings about levee evolution and dimensions, and the implications for geological reconstructions and future delta management.

### Levee development and dimensions

The models show that incipient levee formation occurs when intermediate grain sizes between those of the river channel and those in the distal floodbasin, e.g. silt and fine sands, are transported over the channel where bed shear stress diminishes towards the calm floodbasin (Figure 12a), which proves the hypothesized mechanism in Cazanacli and Smith (1998); Adams *et al*. (2004); Filgueira–Rivera *et al*. (2007); Smith and Pérez‐Arlucea (2008).

We found that levee formation occurs in two phases: an initial and faster heightening phase when levees grow towards the asymptote of water depth, and a slower and more constant widening phase filling the floodbasin. Initially, coarser sediment fractions (e.g. silty sand) are deposited near the channel, contributing to the heightening phase, (i.e. resembling the initial rapid vertical levee growth) (see Figure 4). During the widening phase finer material (e.g. silt) is deposited progressing into clay towards the distal floodbasin. In the absence of these key sediment fractions there is no levee formation and the floodbasin remains relatively flat and filled with clay (Figure 6) without a clear morphological separation from the main channel as in Kleinhans *et al*. (2018). In this case, there is not enough sediment transport from the main channel towards the basin to build levees, even with suitable local bed shear stresses (Figure 12e). From our scenarios we conclude that the necessary conditions to create levees are (1) fluvial‐tidal (overbank) discharge with high water (flood) levels that provide sufficient accommodation space for levee growth and (2) abundant supply of intermediate grain size fractions.

**Figure 11:**
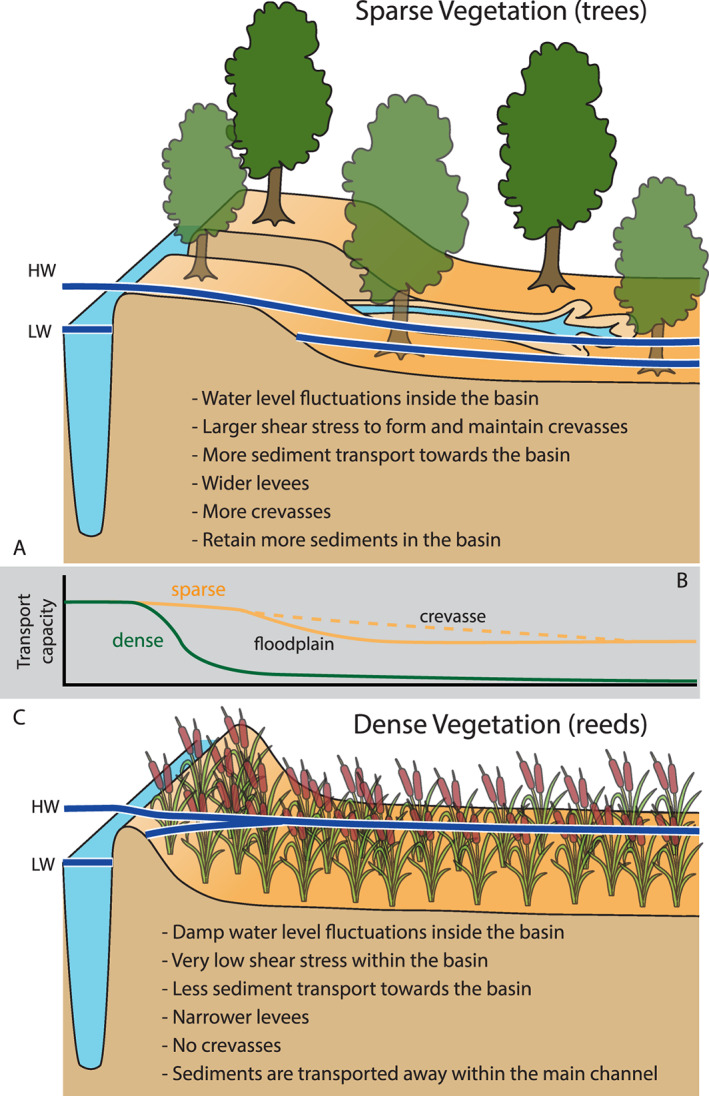
Sketch of levee and floodbasin evolution under fluvial‐tidal boundary conditions with different vegetation typologies: (a) sparse (trees) vegetation and (c) dense (reeds) vegetation. (b) Sketch of sediment transport magnitude across the levee and floodbasin. Vertical dimensions are exaggerated for better visual representation. The minimum and maximum water levels drawn here were largely based on findings from Figure 7c,d. [Colour figure can be viewed at wileyonlinelibrary.com]

**Figure 12:**
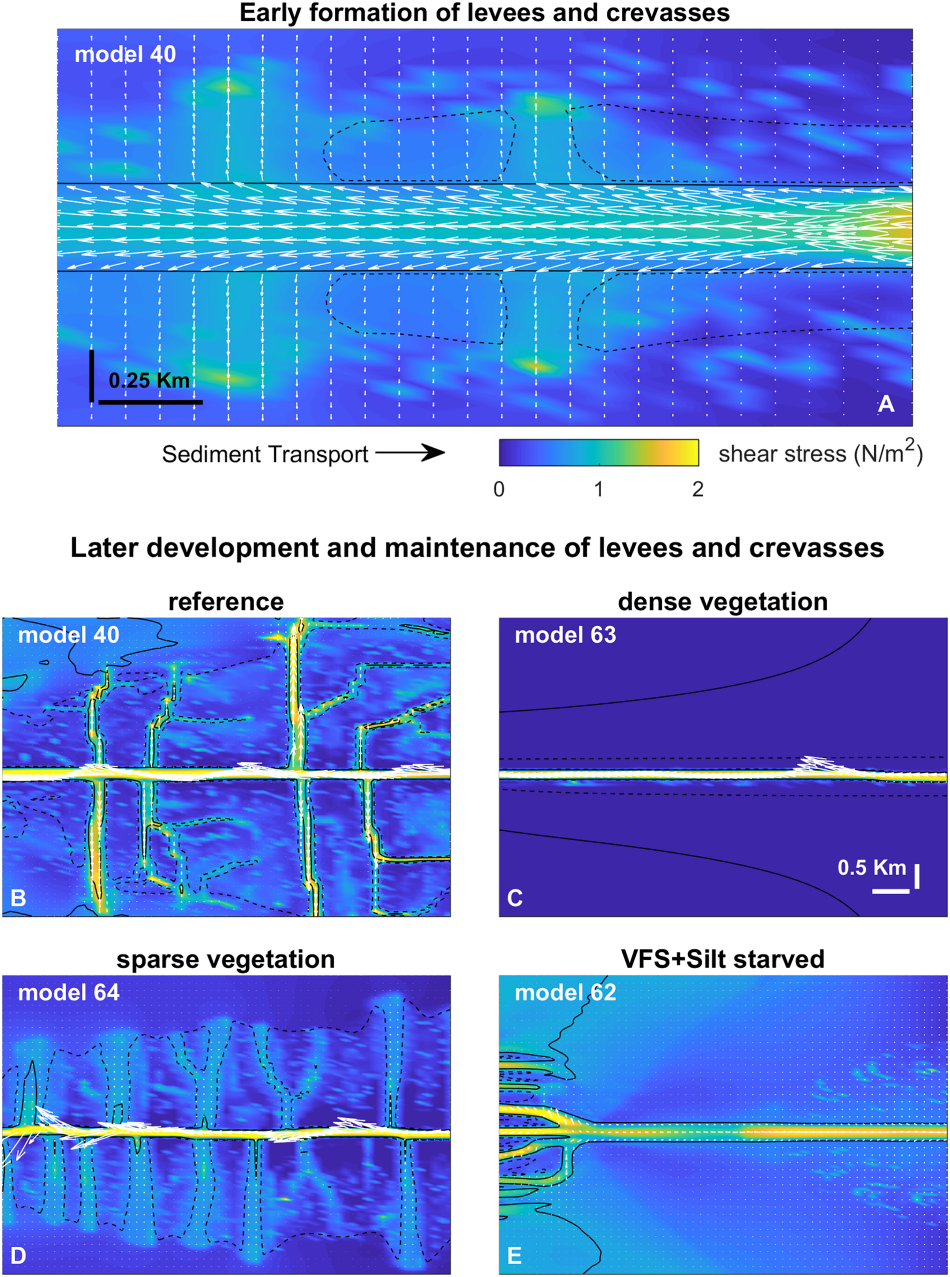
Mechanism of levee and crevasse formation and maintenance. Model scenarios are the same from Figure 6. The plots consist of the maximum bed shear stress over a full tidal cycle (color maps), and tidally integrated (i.e. net) sediment transport (arrows). The channel‐directed (U‐component) sediment transport was reduced to 25% while the basin‐directed (V‐component) was increased by 50% for visualization purposes. The solid black line depicts the depth contour of the main channel and larger breaches, while the dashed lines represent the levees and the smaller breaches. (a) Early levee development from the reference scenario and the simultaneous formation of crevasses. Note the higher bed shear stress focused between the levee sections (dashed lines), and the sediment transport being diverted from the main channel. (b‐e) later stage of levee and crevasse maintenance. These maps are based on the bed evolution stage shown in Figure 6a‐d. [Colour figure can be viewed at wileyonlinelibrary.com]

Levee height depends primarily on the maximum water level induced by tides and floods, given enough sediment availability, while levee width mainly responds to sediment supply (Figure 9). Storms *et al*. (2005); Smith and Pérez‐Arlucea (2008); Esposito *et al*. (2017) demonstrate the importance of river floods in building levees, similarly to our simulations with variable fluvial discharge. In addition, our results demonstrate that vegetation further controls levee dimensions and overall morphology, including the formation and persistence of crevasses (Figure 11). Vegetated levees are higher and narrower than their analogue non‐vegetated scenario (Figures 6,9) as the vegetation reduces sediment mobility and transport (Figure 12b,c), increasing sediment retention (Fagherazzi *et al*. 2012) near the feeder channel. To summarize, levee dimensions derive from the 3‐way interaction between hydrodynamics, vegetation effects, and sediment supply. By changing one of the aforementioned elements the resulting levee will be different in its dimensions and composition, or even nonexistent.

These results partly explain the empirical findings of Cazanacli and Smith (1998); Adams *et al*. (2004), who found strongly varying levee dimensions, without a clear relation between levee dimensions, channel size (or discharge) and sediment composition. We demonstrate that, in addition to these boundary conditions considered by the authors, changes in the hydrodynamics (discharge and water level), vegetation pattern, and sediment supply strongly affect the steady state morphology of levees and crevasses. These extra variables, hardly isolated in field studies but systematically explored in our models, may explain some of the scatter found by previous authors. The scatter in levee dimensions holds specifically for the silty‐rich anastomosing rivers, such as the Columbia and Saskatchewan (Makaske *et al*. 2002), where bifurcations and the division of water and sediment discharge are irregular between the multi‐thread channels (Kleinhans *et al*. 2012), leading to stronger variations in levee morphology.

Water level fluctuations driven by river discharge variations and tides further induce the formation of crevasses. Crevasse channels form through breaches in the levee and may establish a stable crevasse splay system. Crevasse channels diverge and focus the flow away from the main channel, and induce higher bed shear stress and sediment transport through the narrow gaps (Figure 12a). After the incipient stage, the bed shear stress keeps the crevasse opened, whilst delivering sediments further into the floodbasin (Figure 12b,d). As such, crevasses play a complex role: they deliver sediment to the distal parts of the levees and the basin, but also reduce unchannelized flow over the levees that delivers the sediment. The relation between crevasse formation and vegetation is unravelled below and in Figures 11 and 12.

Vegetation strongly determines the formation and persistence of crevasses and crevasse splays (Figure 6). We see that the presence of dense vegetation inhibits tidal propagation and the formation of crevasses, while sparse vegetation triggers more crevasses compared to the reference non‐vegetated case (Figures 6, 7). Both types of vegetation induce larger water level gradients between the channel and floodbasin, however, the dense scenario induced higher water levels in the basin and therefore reduced the bed shear stress and sediment transport capacity from the channel into the floodbasin (Figures 11 and 12c). This combination largely inhibited the formation of crevasses, which was also observed by Nienhuis *et al*. (2018) who modelled single crevasses, and by Mohrig *et al*. (2000) from the rock record. In contrast to dense vegetation, the sparse vegetation created higher water levels in the channel with respect to the basin, maintaining sufficient water flow, shear stress and sediment transport (Figure 12d). This resulted in more crevasses along the levees in comparison with the same scenario without vegetation and dense vegetation. Now we were able to demonstrate the physical mechanism responsible for both the absence, and the inception and maintenance of crevasses. These results are confirmed along the Old Rhine system where wood (sparse vegetation) replaced reed peat (dense vegetation) in the floodbasin (Figure 1f, Stouthamer, 2001; van Dinter, 2013; de Haas *et al*. 2019), that seems to coincide with more crevassing, which is in line with our modelled scenarios. Because of these different tidal basin infilling modes, we conclude that vegetation not only affects levee and crevasses morphology, but also the infilling of an entire estuary. Dense vegetation in the floodbasin particularly reduces the overall tidal prism, basin dynamics, flow velocity, bed shear stress and sediment transport, which inhibits the formation and evolution of crevasses (Figures 7,11,12b‐e) that are efficient sediment conveyors through the basin.

Although we include only two end‐members of vegetation (i.e. dense and sparse) fully covering the floodbasin, this approach clearly shows the isolated effect of these kinds of vegetation found in nature (see Figure 1 and Cazanacli and Smith, 1998; Adams *et al*. 2004; van Dinter, 2013). In reality, vegetation assemblages are patchier and more dynamic, with competition and succession between species (Silvestri *et al*. 2005). We expect that dense vegetation creates higher and narrower levees, which could in turn trigger more crevasses due to the weaker geotechnical nature of such narrow and high levees (analogueous to the superelevation in Mohrig *et al*. 2000). In this scenario, a temporary or local disturbance of vegetation, e.g. studied in Kirwan *et al*. (2008), is likely to initiate a successful crevasse. However, a densely vegetated floodbasin reduces the likelihood of such crevasses being successful as the breach tends to be quickly filled due to the limited water and sediment discharge through the crevasse towards the dense vegetated floodbasin (Figure 12b), as also demonstrated in Nienhuis *et al*. (2018). Other vegetation aspects also affect crevasse formation, avulsion potential and reoccupation of paleochannels, such as perennial versus ephemeral, spatial distribution, and seasonality (see for example  Stouthamer, 2005). This dynamic behavior of vegetation and biota, including settling, growth, organic accumulation, and mortality is beyond the present scope but should be considered in future projects insofar it affects the dynamics of vegetation (Kirwan *et al*. 2008; Marani *et al*. 2013; van Maanen *et al*. 2015; D'Alpaos and Marani, 2016; van Oorschot *et al*. 2017; Kleinhans *et al*. 2018; Lokhorst *et al*. 2018; Brückner *et al*. 2019) and the formation of peat.

Therefore, congruent to observations from Tal and Paola (2007); Davies and Gibling (2011); McMahon and Davies (2018), the character of the vegetation and other biota controls the large scale landscape, for example, between braiding and meandering rivers end‐members. Here we show that vegetation affects levee dimensions, especially width, and the overall levee evolution including the formation of crevasses. The best agreements between measured levees and our models are found when vegetation is included in the modelled scenarios (see Figure 10). The non‐vegetated scenarios create rather wide levees of similar dimensions to rivers much larger than our modelled discharge and sediment load.

### Implications for interpreting geological records

Levees are generally under‐reported in geological rock records (e.g. the discussion in Brierley *et al*. 1997). This is because overbank deposits may extend well beyond the scale of outcrops, which makes it challenging to recognize them in the field. Furthermore, levees have similar lithological signatures as crevasses (splay), and floodbasin deposits, which makes it difficult to identify them in geological records (Brierley *et al*. 1997; Burns *et al*. 2017, 2019). This means that similar lithological signatures identified in the field may have formed under different combinations of fluvial‐tidal boundary conditions, i.e. equifinality (Figures 4, 5). Possible confusion between levee and floodbasin deposits holds especially for more distal levees, where levee deposits grade into floodbasin deposits. Furthermore, levees are acknowledged to have low preservation potential in the geological record (Brierley *et al*. 1997). They can for example be reworked by younger crevasses and other (tidal) channels (see Figure 4 and Burns *et al*. 2019).

The large‐scale effects of vegetation on fluvial‐tidal levee formation identified in this paper are relevant, if not essential, to the reconstruction and interpretation of ancient fluvial‐tidal systems. We see that vegetation dampens the tides and hence controls the tidal influence on the deposits (Figures 6, 7). This implies that, variations in overbank deposits, observed in the geological record, may reflect local, ‘autogenic’ vegetation variations, instead of being the result of changes in boundary conditions offshore or from upstream rivers (Esposito *et al*. 2017; Burns *et al*. 2017). The same is valid for sequences of peat and inter‐fingering crevasse and levee deposits, which might well be self‐organizing features rather than forced by different boundary conditions (in line with e.g. Shen *et al*. 2015). The modelling demonstrated that dense pioneer‐riparian species affect the geomorphology and morphodynamics. Firstly, dense vegetation inhibits levee widening and crevasse formation. Secondly, it can dampen offshore tides to such an extent that the tidal basin transforms to a fluvial‐dominated system (Figure 6b). These findings are summarized and illustrated in Figure 11. This second effect has long‐term consequences for a succession from a tidal environment to a fresh water wetland or peat under constant boundary conditions.

We see these contrasting model scenarios (Figure 6) mirrored in the fate of Holocene Dutch estuaries, some of which filled up and some of which remained open (Vos, 2015; de Haas *et al*. 2018). The key contrasting conditions between tidal inlets on the western coast of the Netherlands, including the Old Rhine, and the Wadden Sea are (1) the history of marine‐fluvial sediment supply, (2) the extent of vegetation and peat, (3) tidal basin shape and orientation affecting local generated wind‐waves (Beets and van der Spek, 2000; Vos, 2015). The factors which causes the difference in morphology is that the Rhine‐Meuse rivers supplied water and sediment discharge to the Old Rhine and Meuse estuary from the landward side (Vos, 2015; de Haas *et al*. 2018; de Haas *et al*. 2019). The freshwater supply was conducive to peat formation. The model results corroborate the theory that sediment supply and vegetation or peat growth are the main causes of infilling in basins and estuaries (Kirwan and Murray, 2007; Esposito *et al*. 2017; de Haas *et al*. 2018; Donatelli *et al*. 2019). On the other hand, the Wadden Sea system had no large sediment input, so the lack of both sediment and vegetation allowed tides and local wind waves to keep it open for longer time periods, similar to e.g. Marani *et al*. (2007); Mariotti and Fagherazzi (2010); Marani *et al*. (2011); Mariotti and Canestrelli (2017); Nardin *et al*. (2018); Deng *et al*. (2018); Donatelli *et al*.(2019).

In summary, the fate of estuaries can be explained by the two‐way interaction of sedimentation facilitating vegetation to settle in shallower grounds and vegetation retaining more sediments. The shallow vegetated areas combined with sediment delivery reduce the hydrodynamics, including the tidal prism and the dissipation of local generated waves. This infilling feedback loop progressively reduces the basin dynamics allowing more deposition (Figure 8).

### Considerations for future delta and estuary management

Understanding the complex interplay between fluvial‐tidal boundary conditions, sediment delivery and the presence of vegetation enlightens the future management of low‐lying areas. For example, the maintenance of water and sediment discharge is essential for infilling the accommodation space created by future expected accelerated sea level rise (e.g. D'Alpaos *et al*. 2007; Kirwan and Murray, 2007). A sediment‐starved system can become subject to positive erosional feedbacks in the presence of tides as the tidal prism grows relative to the accommodation space (Figure 8). Similar evolution has been observed by Marani *et al*. (2007) for the Venice Lagoon along the 20th century and by Eslami *et al*.(2019) on the Mekong Delta. This is an undesirable end situation for low‐lying deltas and estuaries, because it will lead to land drowning. Human interventions such as the construction of artificial levees, river damming, sand mining and water intake, are severe threats to the future sediment budget (Eslami *et al*. 2019; Dunn *et al*. 2019). The combination of reducing water discharge and sediment supply cause many deltas and lowlands to be lost in the near future (Syvitski *et al*. 2005; Nienhuis *et al*. 2020) unless intensive (and expensive) engineering measurements are deployed (e.g. Coastal Protection and Restoration Authority of Louisiana, 2012).

The beneficial effects of vegetation in retaining sediments have been described by (Kirwan *et al*. 2008; Fagherazzi *et al*. 2012; Belliard *et al*. 2016; D'Alpaos and Marani, 2016; Esposito *et al*. 2017). We show how this happens in fluvial‐tidal vegetated environments. Levees and crevasses act as sediment conveyors (Figures 6,11,12 and  Nienhuis *et al*. 2018), while vegetation reduces the tidal prism and thus facilitate more quiet conditions required for sediment deposition (Figure 7 and  Braat *et al*. 2019; Brückner *et al*. 2020). Even when sediment supply is too low to fill basins entirely, the combination of vegetation helping to both fill the accommodation space while also reducing the tidal prism and together with sedimentation corridors (e.g. crevasses) along channels may be an attractive way to distribute sediments that contribute to land rise and enhance flood protection (Zhu *et al*. 2020). When building artificial levees that are never or rarely flooded, most sediments are carried downstream via main channel, and do not contribute to land level rise. Our scenario with dense vegetation and no crevasses also shows that the basin remains rather starved, but levees do develop. Moreover, we expect that organic growth, not incorporated in our model, can still contribute to rise the basin bed level (Mariotti and Canestrelli, 2017).

In summary, the natural or controlled distribution and retention of sediments via the combination of (crevasse) channels and vegetation can inspire the management and nature‐based solutions for drowning coastal landscapes.

## Conclusions

The morphodynamic models presented here show, in isolation and combination, the main effects of fluvial‐tidal boundary conditions, sediment supply and the presence of vegetation in creating levees, crevasses and the overall estuary geomorphic evolution.

Levees grow in height and width when provided with key sediment size fractions, namely, fine sands and silt, (overbank) fluvial‐tidal discharges and sufficient accommodation space. We found that maximum levee height is limited by water level fluctuations induced by tides and floods, while sediment supply and fluvial discharge control their lateral expansion. In general, the combined effects of river and tides create higher and wider levees and trigger more crevasses when compared to fluvial conditions alone. Sufficient sediment supply comprising intermediate grain sizes between those of the river channel and the distal floodbasin are a prerequisite for levee formation.

Our model scenarios demonstrate the mechanisms by which vegetation controls the dimensions and the evolution of levees‐crevasses and hence the overall estuary morphodynamics. In general, vegetation creates higher but narrower levees when compared to the analogue non‐vegetated scenario. Depending on the type of vegetation, the effect of plants on the morphodynamics is to inhibit crevasses (dense vegetation) or to trigger more crevasses (sparse vegetation). In addition, dense vegetation reduces the tidal prism and accommodation space within the basin, ultimately shifting the tidally dominated system towards a fluvially dominant state. Here we showed that even with offshore tidal conditions, the levee fully connected to the barrier island, unlike the other tidal scenarios. This levee connection isolated the floodbasin from the main tidal‐fluvial dynamics. This transition from tidal to fluvial dominated environment was induced solely by the presence of vegetation, allowing levee expansion further downstream, and not by a change in boundary conditions such as tidal or fluvial discharge. Our modelling shows the importance of considering the effects of biota (eco‐engineering species) in geological reconstructions as well as its importance for forecasting future scenarios.

Variable water discharge (e.g. tides and floods) in combination with sediment supply and vegetation is effective in developing levees and crevasses that distribute and retain more sediments into the floodbasin. Especially in tidal‐influenced environments, this is a natural mechanism that fills the accommodation space while reducing the enlargement of tidal prism. This infilling feedback loop is therefore important to keep up with relative sea level rise while preventing the loss of important ecosystems in coastal plains.

## Author Contributions

Conceptualization: Marcio Boechat Albernaz, Harm Jan Pierik and Maarten Kleinhans; Formal analysis: Marcio Boechat Albernaz and Lonneke Roelofs; Methodology: Marcio Boechat Albernaz and Lonneke Roelofs; Numerical modelling: Marcio Boechat Albernaz and Lonneke Roelofs; Field data: Harm Jan Pierik; Writing original draft: Marcio Boechat Albernaz; Review and editing, Lonneke Roelofs, Harm Jan Pierik and Maarten Kleinhans. Funding acquisition: Maarten Kleinhans.

The authors declare no conflict of interest.

## Supporting information


**Supplementary Material 1.** Delft3D MDF, SED and MOR setup used in the reference scenario ID 40.

**Supplementary Material 2.** Levee dimensions (data).
**Supplementary Material 3.** Levee dimensions (models).

## Data Availability

Levee dimensions used in this article are available as supplementary material. Delft3D steering settings from our reference scenario (model 40) are available as supplementary material. Complete data sets and model inputs/results used and/or analyzed during the current study are available from the corresponding author on reasonable request. Delft3D source code is freely distributed and available at the Deltares (SVN) repository: https://svn.oss.deltares.nl/repos/delft3d/tags/delft3d4/7545.

## Appendix A

### Model scenarios

**TABLE A1:** Model scenarios with main boundary conditions and features.

Run	Discharge	Tidal Amplitude	[Mud]	Variations
ID‐	*m*^3^/*s*	[M2‐M4] m	mg/L	‐
38	700	0‐0	20	
39	700	0.50‐0.050	20	
**40**	**700**	**0.75‐0.075**	**20**	
41	700	1.00‐0.100	20	
42	692‐1000	0.75‐0.075	20	
43	678‐1500	0.75‐0.075	20	
44	692‐1000	0‐0	20	
45	400	0.75‐0.075	20	
46	1000	0.75‐0.075	20	
47	1500	0.75‐0.075	20	
48	700	0‐0	10 (only clay)	No silt and VRSand
50	400‐1000	0.75‐0.075	20	
51	700	0.25‐0.025	20	
52	700	1.25‐0.125	20	
53	700	0.75‐0.075	0	
54	700	0.75‐0.075	10	
55	700	0.75‐0.075	30	
56	700	0.75‐0.075	40	
57	685‐1250	0.75‐0.075	20	
62	700	0.75‐0.075	10 (only clay)	No silt and VRSand
63	700	0.75‐0.075	20	dense veg
64	700	0.75‐0.075	20	sparse veg
66	400	0.75‐0.075	20	dense veg
67	1500	0.75‐0.075	20	dense veg
68	678‐1500	0.75‐0.075	20	dense veg
69	700	0.25‐0.025	20	dense veg
70	700	0.50‐0.050	20	dense veg
71	400	0.75‐0.075	20	sparse veg
72	1500	0.75‐0.075	20	sparse veg
73	678‐1500	0.75‐0.075	20	sparse veg
74	700	0.25‐0.025	20	sparse veg
75	700	0.50‐0.050	20	sparse veg
76	400	0‐0	20	
77	1000	0‐0	20	
78	1500	0‐0	20	
79	400‐1000	0‐0	20	
80	678‐1500	0‐0	20	
81	400	0‐0	20	dense veg
82	1000	0‐0	20	dense veg
83	1500	0‐0	20	dense veg
84	692‐1000	0‐0	20	dense veg
85	678‐1500	0‐0	20	dense veg
86	700	0‐0	20	dense veg
87	1000	0.75‐0.075	20	dense veg
88	1000	0.75‐0.075	20	sparse veg
89	400	0‐0	20	sparse veg
90	700	0‐0	20	sparse veg
91	1000	0‐0	20	sparse veg
92	1500	0‐0	20	sparse veg
93	685‐1250	0‐0	20	
94	700	1.00‐0.100	20	dense veg
95	700	1.00‐0.100	20	sparse veg
96	700	1.25‐0.125	20	dense veg
97	700	1.25‐0.125	20	sparse veg
98	692‐1000	0‐0	20	sparse veg
99	678‐1500	0‐0	20	sparse veg
100	1500	1.25‐0.125	20	
101	1500	0.25‐0.025	20	
102	400	0.25‐0.025	20	
103	400	1.25‐0.125	20	
