# Supplementary material for: Natural levee evolution in vegetated fluvial‐tidal environments
Source: Earth Surf Process Landf. 2020 Oct 1;45(15):3824–41. doi: 10.1002/esp.5003 (PMC7780550; doi:10.1002/esp.5003)
Supplement: Supplementary file 1 — Supplementary Material 1. Delft3D MDF, SED and MOR setup used in the reference scenario ID 40. [file ESP-45-3824-s001.docx]

**Supplementary Material 1:** Delft3D MDF, SED and MOR setup used in the reference scenario ID 40.

MDF File:

| Ident = #Delft3D-FLOW 3.59.01.48550# |
| --- |
| Commnt = |
| Filcco = #large_grid.grd# |
| Anglat = 0.0000000e+000 |
| Grdang = 0.0000000e+000 |
| Filgrd = #large_grid.enc# |
| MNKmax = 202 202 1 |
| Thick = 1.0000000e+002 |
| Commnt = |
| Fildep = #depth_012.dep# |
| Commnt = |
| Commnt = no. dry points: 0 |
| Commnt = no. thin dams: 0 |
| Commnt = |
| Itdate = #2000-01-01# |
| Tunit = #M# |
| Tstart = 0.0000000e+000 |
| Tstop = 2.6208000e+005 |
| Dt = 0.5 |
| Tzone = 0 |
| Commnt = |
| Sub1 = # I# |
| Sub2 = # C # |
| Namc1 = #Sediment_1 # |
| Namc2 = #Sediment_2 # |
| Namc3 = #Sediment_3 # |
| Namc4 = #Sediment_4 # |
| Namc5 = #Sediment_5 # |
| Namc6 = #Sediment_6 # |
| Commnt = |
| Wnsvwp = #N# |
| Wndint = #Y# |
| Commnt = |
| Zeta0 = 0.0000000e+000 |
| C01 = 0.0000000e+000 |
| C02 = 0.0000000e+000 |
| C03 = 0.0000000e+000 |
| C04 = 0.0000000e+000 |
| C05 = 0.0000000e+000 |
| C06 = 0.0000000e+000 |
| I0 = 0.0000000e+000 |
| Commnt = |
| Commnt = no. open boundaries: 4 |
| Filbnd = #bnd_def.bnd# |
| FilbcT = #timeseries.bct# |
| Filana = #astr_flow.bca# |
| FilbcC = #transport.bcc# |
| Rettis = 0.0000000e+000 |
| 0.00E+00 |
| 0.00E+00 |
| 0.00E+00 |
| Rettib = 0.0000000e+000 |
| 0.00E+00 |
| 0.00E+00 |
| 0.00E+00 |
| Commnt = |
| Ag = 9.8100000e+000 |
| Rhow = 1.0000000e+003 |
| Tempw = 1.5000000e+001 |
| Salw = 3.1000000e+001 |
| Wstres = 6.3000000e-004 0.0000000e+000 7.2300000e-003 1.0000000e+002 7.2300000e-003 1.0000000e+002 |
| Rhoa = 1.0000000e+000 |
| Betac = 5.0000000e-001 |
| Equili = #N# |
| Ktemp = 0 |
| Fclou = 0.0000000e+000 |
| Sarea = 0.0000000e+000 |
| Temint = #Y# |
| Commnt = |
| Roumet = #C# |
| Ccofu = 5.0000000e+001 |
| Ccofv = 5.0000000e+001 |
| Xlo = 0.0000000e+000 |
| Vicouv = 1.0000000e+000 |
| Dicouv = 1.0000000e+001 |
| Htur2d = #N# |
| Irov = 0 |
| Filsed = #sediment.sed# |
| Filmor = #morph.mor# |
| Commnt = |
| Iter = 2 |
| Dryflp = #YES# |
| Dpsopt = #MAX# |
| Dpuopt = #MOR# |
| Dryflc = 1.0000000e-001 |
| Dco = -9.9900000e+002 |
| Tlfsmo = 6.0000000e+001 |
| ThetQH = 0.0000000e+000 |
| Forfuv = #Y# |
| Forfww = #N# |
| Sigcor = #N# |
| Trasol = #Cyclic-method# |
| Momsol = #Cyclic# |
| Commnt = |
| Commnt = no. discharges: 0 |
| Commnt = no. observation points: 5 |
| Filsta = #obs.obs# |
| Commnt = no. drogues: 0 |
| Commnt = |
| Commnt = |
| Commnt = no. cross sections: 3 |
| Filcrs = #cross.crs# |
| Commnt = |
| SMhydr = #YYYYY# |
| SMderv = #YYYYYY# |
| SMproc = #YYYYYYYYYY# |
| PMhydr = #YYYYYY# |
| PMderv = #YYY# |
| PMproc = #YYYYYYYYYY# |
| SHhydr = #YYYY# |
| SHderv = #YYYYY# |
| SHproc = #YYYYYYYYYY# |
| SHflux = #YYYY# |
| PHhydr = #YYYYYY# |
| PHderv = #YYY# |
| PHproc = #YYYYYYYYYY# |
| PHflux = #YYYY# |
| Flmap = 0.0000000e+000 1000 2.6208000e+005 |
| Flhis = 0.0000000e+000 30 2.6208000e+005 |
| Flpp = 9.7041600e+006 0 9.7041600e+006 |
| Flrst = 144000 |
| Commnt = |
| Online = #N# |
| TraFrm = #vr04.tra# |
| Cstbnd = #yes# |

SED File:

| [SedimentFileInformation] |
| --- |
| FileCreatedBy = Delft3D FLOW-GUI, Version: 3.59.01.48550 |
| FileCreationDate = Fri Jun 22 2018, 11:39:17 |
| FileVersion = 02.00 |
| [SedimentOverall] |
| Cref = 1.6000000e+003 [kg/m3] CSoil Reference density for hindered settling calculations |
| IopSus = 0 If Iopsus = 1: susp. sediment size depends on local flow and wave conditions |
| [Sediment] |
| Name = #Sediment_1# Name of sediment fraction |
| SedTyp = sand Must be "sand", "mud" or "bedload" |
| RhoSol = 2.6500000e+003 [kg/m3] Specific density |
| SedDia = 3.0000000e-004 [m] Median sediment diameter (D50) |
| CDryB = 1.6000000e+003 [kg/m3] Dry bed density |
| IniSedThick = 2.0000000e+001 [m] Initial sediment layer thickness at bed (uniform value or filename) |
| FacDSS = 1.0000000e+000 [-] FacDss * SedDia = Initial suspended sediment diameter. Range [0.6 - 1.0] |
| [Sediment] |
| Name = #Sediment_2# Name of sediment fraction |
| SedTyp = sand Must be "sand", "mud" or "bedload" |
| RhoSol = 2.6500000e+003 [kg/m3] Specific density |
| SedDia = 2.5000000e-004 [m] Median sediment diameter (D50) |
| CDryB = 1.6000000e+003 [kg/m3] Dry bed density |
| IniSedThick = 1.5000000e+001 [m] Initial sediment layer thickness at bed (uniform value or filename) |
| FacDSS = 1.0000000e+000 [-] FacDss * SedDia = Initial suspended sediment diameter. Range [0.6 - 1.0] |
| [Sediment] |
| Name = #Sediment_3# Name of sediment fraction |
| SedTyp = sand Must be "sand", "mud" or "bedload" |
| RhoSol = 2.6500000e+003 [kg/m3] Specific density |
| SedDia = 1.2500000e-004 [m] Median sediment diameter (D50) |
| CDryB = 1.6000000e+003 [kg/m3] Dry bed density |
| IniSedThick = 1.0000000e+001 [m] Initial sediment layer thickness at bed (uniform value or filename) |
| FacDSS = 1.0000000e+000 [-] FacDss * SedDia = Initial suspended sediment diameter. Range [0.6 - 1.0] |
| [Sediment] |
| Name = #Sediment_4# Name of sediment fraction |
| SedTyp = sand Must be "sand", "mud" or "bedload" |
| RhoSol = 2.6500000e+003 [kg/m3] Specific density |
| SedDia = 7.5000000e-005 [m] Median sediment diameter (D50) |
| CDryB = 1.6000000e+003 [kg/m3] Dry bed density |
| IniSedThick = 5.0000000e+000 [m] Initial sediment layer thickness at bed (uniform value or filename) |
| FacDSS = 1.0000000e+000 [-] FacDss * SedDia = Initial suspended sediment diameter. Range [0.6 - 1.0] |
| [Sediment] |
| Name = #Sediment_5# Name of sediment fraction |
| SedTyp = mud Must be "sand", "mud" or "bedload" |
| RhoSol = 2.6500000e+003 [kg/m3] Specific density |
| SalMax = 0.0000000e+000 [ppt] Salinity for saline settling velocity |
| WS0 = 2.5000000e-004 [m/s] Settling velocity fresh water |
| WSM = 2.5000000e-004 [m/s] Settling velocity saline water |
| TcrSed = 1.0000000e+003 [N/m2] Critical bed shear stress for sedimentation (uniform value or filename) |
| TcrEro = 5.0000000e-001 [N/m2] Critical bed shear stress for erosion (uniform value or filename) |
| EroPar = 1.0000000e-004 [kg/m2/s] Erosion parameter (uniform value or filename) |
| CDryB = 5.0000000e+002 [kg/m3] Dry bed density |
| IniSedThick = 5.0000001e-002 [m] Initial sediment layer thickness at bed (uniform value or filename) |
| FacDSS = 1.0000000e+000 [-] FacDss * SedDia = Initial suspended sediment diameter. Range [0.6 - 1.0] |
| [Sediment] |
| Name = #Sediment_6# Name of sediment fraction |
| SedTyp = mud Must be "sand", "mud" or "bedload" |
| RhoSol = 2.6500000e+003 [kg/m3] Specific density |
| SalMax = 0.0000000e+000 [ppt] Salinity for saline settling velocity |
| WS0 = 1.5000000e-005 [m/s] Settling velocity fresh water |
| WSM = 1.5000000e-005 [m/s] Settling velocity saline water |
| TcrSed = 1.0000000e+003 [N/m2] Critical bed shear stress for sedimentation (uniform value or filename) |
| TcrEro = 5.0000000e-001 [N/m2] Critical bed shear stress for erosion (uniform value or filename) |
| EroPar = 1.0000000e-004 [kg/m2/s] Erosion parameter (uniform value or filename) |
| CDryB = 5.0000000e+002 [kg/m3] Dry bed density |
| IniSedThick = 5.0000001e-002 [m] Initial sediment layer thickness at bed (uniform value or filename) |
| FacDSS = 1.0000000e+000 [-] FacDss * SedDia = Initial suspended sediment diameter. Range [0.6 - 1.0] |

MOR File:

| [MorphologyFileInformation] |
| --- |
| FileCreatedBy = Delft3D FLOW-GUI, Version: 3.59.01.48550 |
| FileCreationDate = Thu Jun 14 2018, 09:50:16 |
| FileVersion = 02.00 |
| [Morphology] |
| EpsPar = false Vertical mixing distribution according to van Rijn (overrules k-epsilon model) |
| IopKCW = 1 Flag for determining Rc and Rw |
| RDC = 0.01 [m] Current related roughness height (only used if IopKCW <> 1) |
| RDW = 0.02 [m] Wave related roughness height (only used if IopKCW <> 1) |
| MorFac = 2.0000000e+002 [-] Morphological scale factor |
| MorStt = 7.2000000e+002 [min] Spin-up interval from TStart till start of morphological changes |
| Thresh = 5.0000001e-002 [m] Threshold sediment thickness for transport and erosion reduction |
| MorUpd = true Update bathymetry during FLOW simulation |
| EqmBc = true Equilibrium sand concentration profile at inflow boundaries |
| DensIn = false Include effect of sediment concentration on fluid density |
| AksFac = 1.0000000e+000 [-] van Rijn's reference height = AKSFAC * KS |
| RWave = 2.0000000e+000 [-] Wave related roughness = RWAVE * estimated ripple height. Van Rijn Recommends range 1-3 |
| AlfaBs = 1.0000000e+000 [-] Streamwise bed gradient factor for bed load transport |
| AlfaBn = 1.5000000e+000 [-] Transverse bed gradient factor for bed load transport |
| Sus = 1.0000000e+000 [-] Multiplication factor for suspended sediment reference concentration |
| Bed = 1.0000000e+000 [-] Multiplication factor for bed-load transport vector magnitude |
| SusW = 1.0000000e+000 [-] Wave-related suspended sed. transport factor |
| BedW = 1.0000000e+000 [-] Wave-related bed-load sed. transport factor |
| SedThr = 1.0000000e-001 [m] Minimum water depth for sediment computations |
| ThetSD = 5.0000000e-001 [-] Factor for erosion of adjacent dry cells |
| HMaxTH = 1.5000000e+000 [m] Max depth for variable THETSD. Set < SEDTHR to use global value only |
| FWFac = 1.0000000e+000 [-] Vertical mixing distribution according to van Rijn (overrules k-epsilon model) |
| ISlope = 3.0000000e+000 [-] kf (Koch and Flokstra) |
| AShld = 0.2 |
| BShld = 0.5 |
| NeuBcSand = true |
| Espir = 1 |
| [Underlayer] |
| IUnderLyR = 2 |
| ExchLyr = false |
| TTLForm = 1 |
| ThTrLyr = 0.1 |
| NLaLyr = 0 |
| NEuLyr = 50 |
| ThLaLyr = 0.1 |
| ThEuLyr = 0.1 |
| IDiffusion = 0 |
| Flufflyr = 0 |
| [Output] |
| AverageAtEachOutputTime= true Write mean total transports at each interval to trim-file |
| BedTranspDueToCurrentsAtZeta= true |
